# Biodiversity Trends along the Western European Margin

**DOI:** 10.1371/journal.pone.0014295

**Published:** 2010-12-13

**Authors:** Bhavani E. Narayanaswamy, Paul E. Renaud, Gerard C. A. Duineveld, Jørgen Berge, Marc S. S. Lavaleye, Henning Reiss, Torleiv Brattegard

**Affiliations:** 1 Scottish Association for Marine Science, Scottish Marine Institute, Oban, Argyll, United Kingdom; 2 Institute Akvaplan-niva AS, Polar Environmental Centre, Tromsø, Norway; 3 Netherlands Organisation for Scientific Research, Texel, The Netherlands; 4 The University Centre in Svalbard, Longyearbyen, Norway; 5 Department for Marine Research, Senckenberg Institute, Wilhelmshaven, Germany; 6 Department of Biology, University of Bergen, Bergen, Norway; University of Hull, United Kingdom

The seas along the western European margin encompass a vast geographical area comprising numerous different habitats, and are home to more than 10,000 metazoan species. Although research in this extensive region has been undertaken since the early 1800s, many new species are being described and distributional patterns identified. Recent studies incorporating the most extensive data series ever used in such European studies have failed to find any relationship between latitude and infaunal shelf biodiversity. Along the European shelf, species richness generally increases to a depth of 200 m and then decreases from 300–500 m. In the deep Northeast Atlantic, a unimodal curve illustrates how macrofaunal species diversity changes with depth whilst the megafauna appear to have a bimodal distribution. Regional studies are equivocal in that poleward increases in species diversity have been observed in some studies or taxa, but not in others. In the North Sea, arguably the best studied system in European waters, there appears to be a distinct increase in diversity with increasing latitude. Since this trend is confounded by similar latitudinal gradients in depth and trawling intensity, there is no clear explanation for the biodiversity pattern. Climatic shifts in diversity patterns and species ranges have recently been observed. Here we report previously unpublished data on changes in species richness that have been observed along the Norwegian coast over the past two decades, with the most northerly region seeing more than a 15% increase in the number of species being discovered there. This review synthesizes published and new biodiversity data across multiple spatial and temporal scales, and from the coast to the deep-sea, to provide an overview of what is known along the western European margin. Threats to the biodiversity of the region are highlighted, as well as identifying where there are still gaps in our knowledge.

## Introduction

As anthropogenic disturbance and climate change threaten biodiversity in terrestrial and marine habitats, one of the urgent challenges in ecosystem research is to identify causal links between biodiversity and ecosystem functioning (BEF) and services [1]. Following progress in this field in terrestrial systems [2], [3], many papers have appeared over the last 10 years specifically dealing with marine benthic communities. An important overview of this field is presented in the special issue by Solan et al. [4]. Much of what is known about effects of species richness on ecosystem function comes from relatively small-scale experimentally manipulated systems, where nutrient fluxes were used as a proxy for ecosystem function [5], [6]. Loss or gain of function in these ecosystems was often associated with the loss or gain of key species with specific traits, rather than with species richness per se. Often such key species were ones with a high bioturbation potential [6]–[8], but species with a high growth potential had a similar effect [9]. It has recently been argued that these small-scale studies may in fact underestimate the importance of biodiversity for ecosystem functioning [10]. Examples of BEF studies conducted on much larger scales and involving natural ecosystems in the marine realm are those by Worm et al. [1] and Danovaro et al. [11]. Although such large-scale studies necessarily rely on correlations for inferred causality, the outcomes are in agreement with experimental and theoretical evidence, namely, that high diversity systems provide more services with less variability [1]. Danovaro et al. [11] illustrated this specifically for benthic biodiversity in the deep sea where ecosystem function and diversity are exponentially correlated, implying an exponential loss in function with decreasing biodiversity. It is this developing understanding of the critical role of biodiversity in marine ecosystems that requires a solid understanding of how biodiversity varies in time and space, what mechanisms are responsible, and how human activities may alter present patterns.

### Regional setting

The regional focus of this paper is from ca. 35° N (approximately the same latitude as the entrance to the Mediterranean) to the Arctic and from ca. 25° W (the Mid-Atlantic ridge as the western boundary) to ca. 30° E (the Svalbard archipelago as the eastern boundary), encompassing the shallow and deep waters of the Northeast Atlantic, the North Sea, and the Arctic.

**Figure 1:**
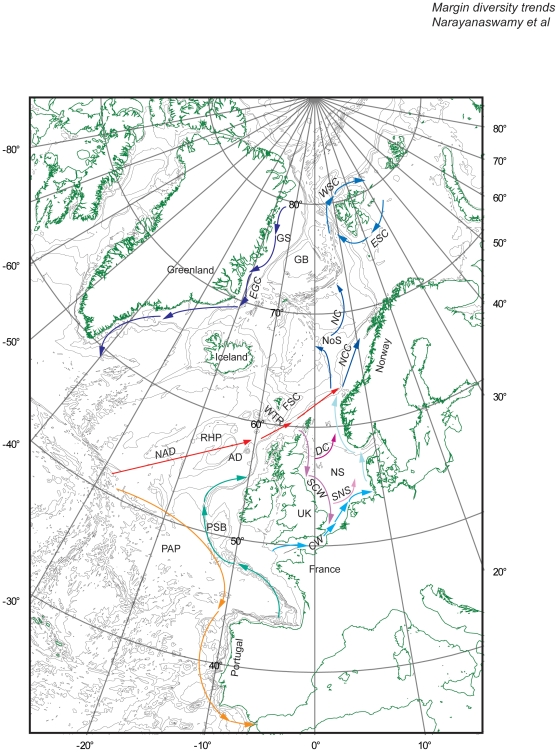
Major seas, topographic features, and surface currents in the area of interest. Acronyms for the seas, topographic features, and currents: GS - Greenland Sea; GB - Greenland Basin; IB - Iceland Basin; RHP - Rockall-Hatton Plateau; PAP - Porcupine Abyssal Plain; PSB - Porcupine Seabight; NS - North Sea; WTR - Wyville-Thomson Ridge; EGC - East Greenland Current; NAD - North Atlantic Drift; SC - Shelf Current; SCW - Scottish Coastal Water; DC - Dooley Current; CNS - Central North Sea Current; SNS - Southern North Sea Current; NC - Norwegian Current; NCC - Norwegian Coastal Current; ESC - East Spitsbergen Current; WSC - West Spitsbergen Current.

The deep-water areas of the Northeast Atlantic (Figure 1) contain many different habitats, including several anomalous shallow plateaus and troughs, such as the Rockall Plateau and Rockall Trough [12]; the vast Porcupine Abyssal Plain; numerous seamounts and canyons; and semi-isolated deep-water basins such as the Norwegian Basin [13], which is separated at depth from the Northeast Atlantic by the Wyville-Thomson Ridge. The Arctic Ocean, on the other hand, may be regarded as a mediterranean ocean, a sea surrounded by landmasses. The ocean itself is not more than about 10 million km^2^, but it has a 45,000 km long coastline, compared with the 112,000 km coastline for the entire Atlantic Ocean. The Eurasian Basin is divided by the Gakkel Ridge to form the Nansen and the deeper Amundsen basins the latter also including the North Pole. Although the basins are relatively deep (maximum depth 5,450 m in the Eurasian Basin), the average depth of the entire Arctic Ocean is not more than about 1,330 m, reflecting the fact that more than half of the area is continental shelf. The North Sea (Figure 1) is part of the European continental shelf sea with depths predominantly from 0–200 m and enclosed by Great Britain, Netherlands, Belgium, France, Germany, Denmark, and Norway. The operational borders of the North Sea are the Dover Strait in the south at 51° N and the 200 m isobath just north of the Shetland Islands at 61°30′ N, where it connects to the Norwegian Sea and the Atlantic Ocean. In the east, the North Sea connects to the Baltic Sea via the Skagerrak and Kattegat between Denmark, Norway, and Sweden. The North Sea is more than 970 km long and 560 km wide, with an area of about 750,000 km^2^.

### Hydrography

#### North Atlantic and Arctic oceans

One of the most conspicuous and important factors influencing marine ecosystems in the North Atlantic and the Arctic is the Gulf Stream System (GSS). Together with its northern extension toward Europe, the North Atlantic Drift (NAD), the GSS is a powerful, warm ocean current that originates in the Gulf of Mexico, follows the eastern coastlines of the United States and Newfoundland, and crosses the Atlantic Ocean toward Northwest Europe. The Gulf Stream not only dramatically warms the climate of countries such as Norway and Great Britain but also has a major influence on the marine life in this region. The warm water of the NAD mixes with the colder waters found in the North Atlantic, increasing turbulence and thus nutrient availability, which in turn resulted in some areas of the North Atlantic becoming the most productive fishing grounds in the world, until overfishing led to a dramatic decline (see the Census of Marine Life project History of Marine Animal Populations [HMAP] for more information www.hmapcoml.org). These large-scale circulation patterns structure the marine (and terrestrial) climate and habitats of the entire area and extend across more than 60 degrees of latitude, and are therefore a major consideration when looking at potential latitudinal gradients in biodiversity. Our study area is largely influenced by the NAD and its northern extension, the West Spitsbergen Current (WSC), along with water exiting the Arctic throught the Fram Strait as the East Greenland Current (EGC) and coastal waters modified by their respective nearshore processes (e.g. waters exiting the North Sea). This leads to dramatic spatial gradients on a variety of scales with potentially strong, but poorly studied, impacts on macrofaunal diversity patterns.

#### North Sea

The North Sea has two major inputs of Atlantic water driven by ocean tidal motion - one in the south through the English Channel and Dover Strait and one in the north along the Shetland Islands. Most of the northern input circulates north of Dogger Bank counterclockwise through the central and northern North Sea. Along large parts of the Scottish and English coasts, there is a southward flow of northern coastal water entering the southern North Sea at 53° N. The Atlantic water entering the southern North Sea through the Channel follows a northward direction, parallel to the coasts of Holland, Germany, and Denmark. But close to the coast, input by large rivers (Rhine, Meuse, Elbe) gives rise to a distinct coastal water mass with low salinity and high turbidity. Seafloor topography largely drives water circulation within the North Sea. The combination of current speed, depth, wave height, and density structure control the heat transfer through the water column and, consequently, stratification. In addition, borders (fronts) between thermally stratified and mixed areas play an important role in the ecology of the North Sea. The presence or absence of stratification also has major consequences for the temperature regime near the bottom and notably the maximum temperatures in summer. Bottom water temperatures in the southern North Sea vary on average between 5°C and 16°C, with more extreme values in shallow coastal waters. These temperatures are relatively high with respect to latitude because of the combination of depth and water supplied by the warm NAD. In the northern North Sea the temperature range is much smaller, with a variation between 6.5°C and 9°C.

### History of research

Systematic study of the North Sea fauna has a long history dating back to inventories of the tidal zone. The successful use of dredges by Audouin and H. Milne-Edwards in France (1826–28) and Michael Sars in Norway (1829) opened the way to study the deeper waters of the North Sea. From 1839 onward the British began to systematically investigate their marine fauna, and Edward Forbes was one of the main instigators. Results of dredging campaigns led in 1859 to Forbes's book on the *Natural History of European Seas*, in which he divided the European seas into so-called faunal provinces on the basis of the benthic fauna. Because of the economic importance of fisheries, scientific institutions for fishery research were founded around the North Sea, for example, in Germany in 1885 and in the Netherlands in 1888. Their scientific activities also led to more knowledge about the invertebrate fauna [see 14, 15], as this was recognized as a main food resource for fish.

In founding the International Council for the Exploration of the Sea (ICES) in 1902 the participating countries (Norway, Sweden, Denmark, Finland, Russia, Great Britain, Germany, and the Netherlands) were committed to regular surveys of the fish fauna in the Northeast Atlantic. This resulted in more vessels becoming operational and more data on various aspects of the North Sea ecosystem becoming available.

At the end of the nineteenth century, the first marine laboratories were founded. In Scotland in 1884, laboratories were opened in St. Andrews and Granton, the latter being the precursor of the Scottish Marine Biological Association (1897) in Millport, now the Scottish Association for Marine Science in Oban. A second laboratory was opened in the UK, the Plymouth Marine Laboratory (1888) following the foundation of the Marine Biological Association of the United Kingdom (1884). In the Netherlands, the Zoological Society (1872) erected a mobile station in Den Helder (1876) that became the precursor of the Netherlands Institute for Sea Research, now based at Texel. The first complete survey of the North Sea was only conducted in 1986 by a concerted action of the North Sea Benthos Group of ICES [16]. Several countries around the North Sea, through national monitoring programs, have continued to undertake surveys of their regions of the North Sea.

The Northeast Atlantic deep sea is one of the most well studied deep-water areas in the world and has often been described as the cradle of deep-sea biology [17]. Samples collected by the *Lightning* and *Porcupine* expeditions in 1868-70 highlight the historical importance of this area [18]. Subsequently there have been numerous other deep-water expeditions to the European part of the Northeast Atlantic, both organized by individual European countries and as joint European investigations. In the early 1970s, long-term temporal studies of the fauna were undertaken in the Rockall Trough to investigate reproduction and growth [19] as well as map the distribution of megafauna with respect to hydrography and bathymetry [20]. Some of the more recent studies funded by the EU include BENGAL (High-resolution temporal and spatial study of the BENthic biology and Geochemistry of a northeastern Atlantic abyssal Locality), OASIS (OceAnic Seamounts: an Integrated Study), HERMES (Hotspot Ecosystem Research on the Margins of European Seas), which is associated with the Census of Marine Life Continental Margin Ecosystems on a Worldwide Scale (COMARGE) program, and MarBEF (Marine Biodiverstiy and Ecosystem Functioning).

One of the primary goals of the MarBEF program was to compile existing data and facilitate analyses so a more holistic view of biodiversity in European waters can be obtained. The initial effort focused on soft-sediment benthos and resulted in the “Macroben” database [21] from 44 contributed datasets consisting of more than 465,000 records. These records span more than 22,000 sampling stations from the Mediterranean and Black Seas to Franz Josef Land, and include 7,203 valid taxa [21]. Efforts are under way to compile databases for hard-bottom invertebrates and for nematodes. Initial analyses on the Macroben database have tested classification systems for biogeographic zones, investigated spatial trends in biodiversity and community assembly processes, and applied macroecological tools to marine benthos.

The Census of Marine Life (Census), European Census of Marine Life (EuroCoML), and affiliated programs have been extremely active in the Northeast Atlantic, North Sea, and Arctic region. There have been a variety of Census field projects operating in the areas of interest, looking at a number of different environments ranging from the coastline down to the deep sea. An important aspect of the program is to ensure that all the data collected are housed in a central location and preserved for future reference. There is a dedicated online open access system within Europe, EurOBIS, which like the Ocean Biogeographic Information System, not only houses new data that have been collected but aims to collect and store historical datasets from around Europe.

EuroCoML decided that four separate review papers were needed to cover the vast area of European Seas. The regional focus of this paper is from about 35° N to the Arctic and from about 25° W to about 30° E, encompassing the shallow and deep waters of the Northwest European margin, the North Sea, and the Arctic. Separate reviews relating to marine biodiversity in the Baltic Sea [22] and the Mediterranean [23], [24] can be found elsewhere in this collection. Against this backdrop of different environments, our specific aims were to summarize a) spatial patterns of soft sediment benthos and fish, b) regional patterns of biodiversity with regard to latitude and depth, c) shifts in species distribution, regime shifts and anthropogenic impacts, and d) synthesise the new results and findings in a novel way and to add value to the research already undertaken.

### Sampling European marine biodiversity

Generally, biodiversity estimates of soft-sediment macrofauna in shallow-water areas are based on counts of species or higher taxa in discrete grab or core samples after sieving over a mesh size of usually 1 mm. Over the past century, many types of grabs and corers have been devised, and the most common ones used today are the van Veen grab and the Reineck boxcorer [25]. The choice of grab and sieve size can have important implications for biodiversity estimates, as some grabs are known to be inefficient in collecting deep-living macrofauna species [26] such as the common thalassinid crustaceans in the North Sea. Similarly, using too large a mesh size leads to loss of small macrofaunal species like interstitial crustaceans and polychaetes, which can be abundant and species-rich in coarse sediments. In practice, choice of equipment is often a compromise based on size of the ship and time constraints. For sampling mobile epibenthos, an even wider variety of gear has been used, ranging from commercial fishing otter trawls [27] to custom-made beam trawls of different dimensions [28], [29]. The routine use of depth sensors attached to the beam trawl in the most recent surveys has enabled an estimation of the trawled seabed surface and has thus provided semiquantitative epibenthos data. Nevertheless, the efficiency of different designs has only been tested in a few cases and has revealed large errors, depending on species, substrate, and rigging [28], [30].

The collection of macrofauna from the deep waters of the Northeast Atlantic is undertaken using different types of corers, namely, box corers [31] or some form of multi/mega corer [32]. Currently, there is much discussion about the use of different corers. The box corer is thought to undersample the fauna because it creates a bow wave, thus “blowing away” the light-weight fauna living on the sediment surface [33]. The sediment is sieved through mesh sizes ranging from 0.25 to 0.5 mm for macrofauna. However, there is still considerable discussion among scientists within Europe regarding what mesh size to use; researchers in different countries use different mesh sizes and this makes it difficult to compare data. We suggest that in order to make comparisons between shallow water and deep water samples, a series of stacked sieves ranging from 1 mm through to 0.25 mm be used, thus allowing for different data sets to be compared. Furthemore we suggest that deep-water macrofaunal samples should be routinely collected on a 0.25 mm mesh and in shallow water regions, a 1 mm mesh is probably sufficient. However, this does depend on what the purpose of the study is. If it is to record biodiversity then a finer mesh sieve should be used (for reviews see [34], [35]).

Another problem to be considered is whether the samples should be fixed in formalin prior to, or after, sieving. Sieving after fixation is thought to lead to i) a reduction in the number of animals lost and ii) more intact specimens [36]; However, sieving prior to fixation results in smaller quantities of formalin being used and lower sample storage requirements on board ship. A technique that is currently being used by some deep-sea researchers is elutriation. Rather than washing the sediment sample directly on a sieve, water is bubbled through a flask (only feasible for small quantities of sediment) or bucket (for larger quantities of sediment) and the water-sediment mixture is allowed to gently overflow on to a sieve (see [37]). The fauna collected using this technique are often still alive and in one piece which greatly aids identification.

### Measuring biodiversity

How to measure biodiversity is the subject of many past and ongoing scientific discussions involving issues of scale and organization level (genotypes, species, higher taxa, habitat, ecosystem) [e.g., [38]–[41]]. Margurran [41] has reviewed many of the indices that are currently used and highlights the advantages and disadvantages in using those that are currently favored. A diversity index may often continue to be used despite inherent biases as researchers wish to be able to compare results from their studies with others. A way of overcoming this would be to continue to use the old index, but also start using newer indices that have less bias associated with them. An index that is becoming more commonly used is Clarke and Warwick's taxonomic distinctness index [42]–[44] as it has the added advantage of not being as sensitive to sampling effort [45]. However, comparisons across assemblages can be problematic [46]. An in-depth discussion regarding whether one index is better than another e.g. whether Shannon's species diversity is better than evenness or delta plus falls outside the scope of the present paper, which focuses on estimates of biodiversity in the North Sea, Northeast Atlantic, and Arctic. In this paper, we summarize results from studies that have solved this problem using different techniques, depending on the specific research questions addressed and available data. For the most part, we focus on results concerning species richness (total species counts, S), and Hurlbert's rarefaction, ESn. ESn represents the expected number of species when *n* individuals are randomly drawn from the sample. For example, ES50 is the number of species expected to be found in 50 randomly selected individuals from a sample. Hurlbert's index is one of the most commonly used indicies in benthic communities, where samples are of an uneven size, and thus is appropriate for the continental- and regional-scale comparisons we make here, even though it may over-estimate the number of species present [47].

## Results and Discussion

### European-scale spatial patterns

Due largely to local and regional environmental monitoring efforts, nearshore benthos (littoral to shelf break) is the best studied component of European marine biodiversity. Many of these studies cover a relatively small scale, but recent integrative efforts by MarBEF have resulted in analysis of data compiled from across the European seas, and it is estimated that there are more than 10,000 species in the areas of interest (Table 1 [48]). Spatial patterns in biodiversity related to latitude and depth have received the greatest attention, but other physical or ecological gradients such as temperature, sediment grain size and fresh water input, have also been addressed in the regional studies, and may help to suggest mechanisms responsible for generating and maintaining patterns.

**Table 1:** Taxonomic classification of species reported along the Western European margin.

Taxonomic group	No. species	State of knowledge^1^	No. introduced species	No. experts	No. identification guides
**Domain Archaea**	∼	2	∼		∼
**Domain Bacteria (including Cyanobacteria)**	∼	2	∼	∼15	<5
**Domain Eukarya**					
**Kingdom Chromista**	1642	2	5	∼8	>5
Phaeophyta	∼			∼5	
**Kingdom Plantae**					
Chlorophyta	518	3	5	∼10	>5
Rhodophyta	1257	3	25	∼5	>5
Angiospermae	∼			∼20	>5
**Kingdom Protoctista (Protozoa)**					
Dinomastigota (Dinoflagellata)	444	3	10	>5	<5
Foraminifera	>5	3		>5	<5
**Kingdom Animalia**					
Porifera	462	4		>20	>5
Cnidaria	487	4	15	>20	>5
Platyhelminthes	251	4	6	>15	>5
Mollusca	1304	5	55	>50	>10
Annelida	1554	5	15	>50	>10
Crustacea	2244	5	61	∼60	>10
Bryozoa	339	5		>10	>5
Echinodermata	291	5		>10	>5
Urochordata (Tunicata)	102	4	9		>5
Other invertebrates					
Vertebrata (Pisces)	1148	5	39	>15	>10
Other vertebrates	222	5		>10	>5
**SUBTOTAL**					
**TOTAL REGIONAL DIVERSITY**	**12269**		**∼245**		

**Note:**

1 State of knowledge, key:

5  =  very well known (>80% described, identification guides <20 years old, and good taxonomic expertise).

4  =  well known (>70% described, identification guides <50 years old, good taxonomic expertise).

3  =  poorly known (<50% species described, identification guides old or incomplete, moderate taxonomic expertise).

2  =  very poorly known (only few species recorded, no identification guides, little taxonomic expertise).

1  =  unknown (no species recorded, no identification guides, no expertise).

Earlier studies have noted several phenomena pertinent to our region of interest. First, fewer species are recorded in the Arctic than in boreal areas [e.g. 49]. Second, the Arctic is a relatively young habitat in evolutionary terms [50], [51], and hence holds few endemic species within some taxonomic groups. This is particularly true for Arctic shelf areas, because of pronounced sea-level variations during the glacial and interglacial periods [52], but taxonomic distinctness has also been shown to increase across the entire region at depths less than 200 m [53]. Large parts of the European Arctic and North Atlantic region are strongly influenced by warm-water currents coming from the south by means of the northern branches of the GSS. The highest species diversity occurs in the Norwegian and Barents Seas, perhaps due to this influx of water and organisms from the North Atlantic [53]. Species richness decreases northward and eastward into the eastern Barents, Kara, and Laptev Seas [54] (see below).

Systems of geographical delineation of faunal regions may be useful both for investigation of underlying ecological or environmental processes, and for developing meaningful management policies at different spatial scales. While multiple systems of drawing biogeographical boundaries have been proposed, empirical data have rarely been sufficient to test their rigor. Arvanitidis et al. [55] used a dataset containing 5,012 species extracted from Macroben to test the validity of eight systems, and found that polychaetes were the only faunal group to sufficiently support any of the main biogeographical systems, and this was the one proposed by Longhurst [56]. The polychaete group is the third most species-rich in Macroben, has high functional diversity, and is the only macrofaunal group that exhibits all major feeding strategies such as carnivore, surface deposit, sub-surface deposit, filter and inter-face feeding. These features make polychaetes well suited for evaluating the Longhurst biogeographical system, one based largely on water-mass and plankton-community distributions.

Two of the more prevalent biodiversity patterns described in the literature are the decrease in richness with increasing latitude (latitudinal species-diversity gradient, LSDG [e.g., [57], [58]]) and unimodal pattern of richness with water depth, peaking at around 2,000 m [e.g., [59], [60]]. While recent meta-analytical methods have compiled significant quantities of data to evaluate LSDG [61], [62], most of the basis for these patterns in the marine system is from relatively few stations, or includes potentially confounding factors, such as varying sampling effort or covariates. Few marine sampling programs have been designed to test large-scale latitudinal patterns, and the total area of the seafloor sampled is, in many areas along the latitudinal gradient, far too small to feel confident that we have a reasonable value for regional or continental diversity, even in European waters where sampling has been among the most intense.

Two approaches used Macroben to evaluate biodiversity patterns across the whole of European waters, albeit restricted to continental shelf (0–475 m) depths. Whereas an increase in richness to 200 m depth was observed in both studies, no evidence was found to support a LSDG once sampling effort (number of stations, area sampled) and depth covariates were removed [63], [53]. Results were consistent whether the entire community was taken together or major taxonomic groups were analyzed separately, and mollusks even showed a small increase in diversity with increasing latitude [53]. In addition, there was some indication that species richness was (negatively) related to the (modeled) amount of organic matter reaching the seafloor [63]. Each of these studies used subsets of Macroben (extracted so data were collected by comparable methods) that contained well over 3,000 samples and 2,200 species, making them among the largest empirical datasets used to test these patterns.

Novel statistical techniques were used to explore mechanisms that may generate the observed patterns. Processes invoking isolation of subpopulations and subsequent expansion from multiple refuges were more strongly supported than expansion from a center of origin [55]. Additionally, it appears that regional processes determine community assembly for most subsets of the data, whereas random assembly, followed by local environmental and ecological processes, appears to be more important for polychaetes [64]. These efforts begin to bridge the gap between describing pattern and identifying mechanism.

The use of new mathematical models to predict species richness based on sampling intensity and knowledge of habitat characteristics offers promise for both research and management of European biodiversity. Mid-domain effect modeling [65] links species range distributions within a defined domain to predict spatial patterns of biodiversity. Further, ecological knowledge of habitat-species diversity relationships can be combined with remote sensing to predict diversity patterns over large spatial scales when sampling is logistically difficult [e.g. [66], [67]]. These models can be run in a forecasting mode to predict, for example, the effects of habitat loss, homogenization, or fragmentation on biodiversity [67]. Finally, spatial habitat modelling uses a limited set of environmental parameters (e.g. sediment grain size, bathymetry, tidal currents) that describe geomorphological features to predict distributions of marine benthic communities [68]–[70].

Standard ecological questions are often best addressed by studies across small- or regional-scale gradients. Macroecology, however, asks questions on large and multiple scales, often spanning several ecosystems [71]. This offers a complementary approach, and one that is valuable since impacts of climate change and human activities often express themselves on these scales. Because macroecology in its recent formulation is a relatively new discipline [e.g., [72], [73]], it is probably not surprising that the principles have not received much attention in the marine realm. The establishment of Macroben allowed one of the first attempts to search for consistent, large-scale relationships between abundance and distribution of soft-sediment benthic organisms on a pan-European scale. Webb et al. [74] showed that assemblages exhibited the same strongly right-skewed frequency distribution observed in many terrestrial systems; that is, most species are rare and only a few are widely distributed. This also complies with the general understanding that most marine sediment habitats exhibit high spatial heterogeneity in environmental characteristics on many scales. Whereas the general positive relationship between abundance and occupancy shown by Macroben assemblages was similar to that found in many other systems, this relationship was weak, and the sign and strength varied among taxonomic groups and regions within the European domain [74]. Whether this is due to human-induced disturbance patterns, undersampling of the fauna (leading to a suggestion of a greater proportion of rare species than actually exists), or ecological (e.g., life-history) processes remains to be determined. These results, however, show the value of applying new tools and a macroecological perspective to biodiversity research.

### Regional patterns in biodiversity

#### Latitudinal gradients

Interest in large-scale patterns of benthic communities and their biodiversity was stimulated around 1986 by the growing recognition that the intensive bottom- and beam-trawl fisheries in the North Sea were posing a threat to benthic communities, biodiversity, and habitat. Subsequent investigations such as IMPACT have largely confirmed these negative trawling effects [75]. The 1986 NSBS survey yielded the first comprehensive dataset of macrobenthic species and community distribution in the southern and central North Sea [16] and showed that macrobenthos biodiversity expressed as either sample species richness or ES50 increased in a south-to-north direction [76]. This increase in species number was most prominent between 51° N and 58° N. In the deeper central North Sea north of Dogger Bank, where sediment becomes silty-fine sand, species richness further increases up to 58° N, which roughly coincides with the 100 m isobath. North of 58° the latitudinal increase in species richness apparent in the NSBS dataset seems to level off [76]. In a later analysis of the same NSBS dataset, Heip and Craeymeersch [77] showed that the latitudinal increase of macrobenthic diversity holds for all four major taxonomic groups and that this trend contrasts with the diversity of meiofaunal copepods, which shows the opposite pattern, that is, a decrease from south to north.

In an earlier analysis of the NSBS data from the southern and central North Sea, Duineveld et al. [78] linked species richness with assemblage type, instead of latitude. They showed that species richness differed among the different assemblages present, the assemblages in turn being strongly linked to sediment grain size. Fine-grained sediments with moderate amounts of silt had, on average, higher species richness than mobile sandy sediment. This difference can be partly explained by the extensive three-dimensional subsurface structures formed by animal burrows in this type of sediment (e.g., thalassinid shrimps, echinoderms), increasing heterogeneity and complexity [see 79, 80], and the moderate intensity of physical disturbance [81]. However, neither sediment nor assemblage type completely explains the gradient. On the shallow Dogger Bank north of the Oyster Grounds, with relatively uniform sandy sediment and inhabited by an assemblage similar to the southern North Sea, species richness is equally high as in the Oyster Grounds [see 82]. According to Kröncke [83], the relatively high species richness on Dogger Bank is possibly a development of the last 50 years. As no earlier data exist for other areas, it is impossible to say if the whole gradient is likewise a recent development.

**Figure 2:**
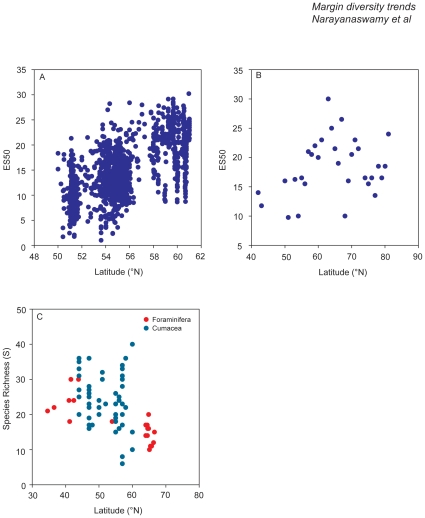
Changes in species diversity with latitude. (A) The ES50 for the North Sea soft-sediment benthos. The figure shown here excludes the data from the English Channel results and thus has been re-analysed and re-drawn to take this fact into account (see Rees et al. [84] for the full analysis). (B) The ES50 for the soft sediment European continental shelf macrofauna – mean values at 1° latitudinal bands (adapted from Renaud et al. [53]). (C) The species richness for the Foraminifera and Cumacea found in the deep Northeast Atlantic (Foraminifera adapted from Culver and Buzas [110]; Cumacea adapted from Gage et al. [105]).

Recently an effort has been made to compare patterns found in the 1986 NSBS survey with newer data from 2000–2001 [84], but has to now not been published in the peer-reviewed literature. In contrast to the synoptic grid data from 1986, the 2000 dataset was assembled from different national surveys, which led to increased spatial coverage. Nearly all the component surveys from 2000/2001 that have been included in these analyses were performed with the same sampling gear i.e. 0.1 m^2^ van Veen grab and a 1 mm mesh [84]. Here we have undertaken further analysis, by focusing specifically on the North Sea data (2000–2001 included results from the English Channel). The ES50 results collected from 14 datasets utilising only the North Sea data clearly illustrates that macrobenthic biodiversity increases in a northerly direction (Figure 2a). Nevertheless, the same trend in species richness ES50 found in 1986 was also very clear in the combined 2000 data and the North Sea data (used here), with no significant difference between absolute numbers [85]. The stability of the diversity pattern is also illustrated in Daan and Mulder [82], who showed that differences in species richness among four assemblages in the Dutch part of the southern North Sea were constant over the period 1991–2005. Changes in species richness over the period 1991–2005 were only observed in the assemblage inhabiting the muddy and summer-stratified Oyster Grounds, where an increase was seen. No obvious environmental factors explained this trend [86]. On a smaller local scale, effects of environmental variability on species richness have been well documented [87]–[89]. In the coastal reaches of the German Bight, severe winters have distinct impacts on species richness [30]. Furthermore, nutrient concentrations and river runoff have been found to correlate with species richness in the German Bight [90].

Discriminating which variable is responsible for the observed richness trend, given that a single variable exists, is problematic considering that most variables are correlated (e.g., wave stress and depth, stratification and temperature). Nevertheless, the common message in the papers above is that hydrographical variables strongly influence the latitudinal trend in macrofauna species richness, and bottom water temperature seems the most influential. One factor not accounted for in explorations of latitudinal diversity trends in the North Sea is the effect of trawling, and especially beam- trawling, on benthos. Intensity of beam trawling is highest in the southern North Sea [see 91], where sandy sediments have relatively low species richness. Whether this is a causal link has not been verified. Models predict low impact of trawling in habitats with a high degree of natural disturbance, such as mobile sands [91], suggesting that the low species richness might be a natural feature of the habitat.

Quantitative investigations of soft-sediment epibenthos for the entire North Sea were undertaken, initially by Frauenheim et al. [92], and then continued by Jennings et al. [29], Zühlke et al. [93], and Callaway et al. [94] using standardized gear and sampling methods. However, detailed analyses of diversity patterns of epibenthos were hampered by the still variable trawling distance per haul, and the low and partly unknown catch efficiency of the beam-trawl. Nevertheless, the observed large-scale spatial patterns of epibenthic species diversity were similar to patterns in the infauna, showing a clear latitudinal trend, with lowest diversity in the southern and highest in the northern North Sea. This gradient was even more conspicuous for sessile epibenthic species. Particularly at depths between 50 m and 100 m, a diverse sessile fauna was found, dominated by hydrozoans and bryozoans, whereas along most parts of the continental coast, total species diversity was rather low [94]. Epibenthic diversity plotted against latitude produced a clear relationship with latitude, increasing from south to north [95].

On the European continental shelf, LSDGs of soft-sediment fauna have been studied over a range of scales, from 15 to 45 degrees of latitude, and with variable results. At distance ranges of about 15 degrees in the Arctic Ocean, diversity of macrofauna and nematodes was found to decrease with increasing latitude, after controlling for depth (Figure 2b) [95], [96]. Conversely, Ellingsen and Gray [79] found no evidence for an LSDG over a range of 15 degrees along the Norwegian coast. Finally, some of the most well known examples of diversity gradients in European waters come from the Baltic, where there is a strong decline in diversity with latitude [e.g., 97], and the North Sea, where the trend is reversed and diversity increases with latitude [76], [84]. More detailed discussion of the North Sea and Baltic Sea datasets is provided either earlier in this review or in a companion paper in this collection, respectively [22]. These widely varying results over smaller spatial scales suggest that local and regional processes are important in determining spatial patterns of infauna and epifauna, whereas at continental scales (up to 45 degrees of latitude) there is no obvious support for LSDGs (see discussion above).

Fish species richness has been shown to decline with increasing latitude both globally [62] and within the European region [61]. Assessment of these patterns, however, is only as good as our current estimates of local and regional biodiversity. A recently completed seven-year study led to an increase of more than 10% in the number of Europe's freshwater fish species [98]. These results from a rather well studied habitat (Europe's freshwater) suggest that it is likely that concentrated sampling of marine fish in poorly known areas of Europe's coastal and deep-sea zones, and in Arctic areas in particular, may result in comparable changes in our understanding of fish biodiversity and perhaps in latitudinal trends in that diversity.

There has been an order of magnitude fewer studies examining deep-sea diversity latitudinal gradients. Both ecological and evolutionary processes appear to influence global-scale deep-sea diversity patterns, although the patterns themselves are still not fully understood. In studies investigating the deep waters of the Northeast Atlantic and up into the Arctic, there does not appear to be a strong latitudinal diversity gradient. The Greenland-Iceland-Faroe Ridge acts as a physical barrier and thus has an effect on the overall diversity. Work published by Russian researchers in the late 1980s found that the species richness of many different faunal groups, including cumaceans [99], echinoderms [100], and prosobranch gastropods [101], was much lower in the Arctic deep sea than on the nearby shelves.

More recent large-scale studies and analyses have investigated the influence of latitude on diversity, most notably Rex et al. [102]–[104] looking at deep-sea bivalves, gastropods, and isopods and Gage et al. [105] looking at Cumacea in the deep Atlantic. Lambshead et al. [106], [107] and Mokievsky and Azovsky [108] investigated the links between nematodes and diversity gradients while Thomas and Gooday [109] and Culver and Buzas [110] looked at foraminifera. Rex et al. [102] found that there was a poleward decline in deep-water molluscan and crustacean diversity in the Northwest Atlantic. Gage et al. [105] also found that for the cumaceans there was a poleward decline in diversity in the Northeast Atlantic; with a steeper regression line in the east compared to the west side of the basin. When Gage et al. [105] included samples from the Nordic Seas, they found that the slope of the regression line markedly increased. Lambshead et al. [106], however, did not find a decline in nematode diversity associated with latitude; instead they reported a small positive gradient that they attributed to increasing surface productivity. Culver and Buzas [100] and Corliss et al. [111] found that foraminiferal diversity in the North Atlantic also exhibited a poleward decline and attributed this to trends in food supply. Here we have combined Gage et al's. [105] cumacean results (minus those from the Nordic Seas) with the foraminiferan results [100], and as can be seen the decline in cumaceans is not so clear as is with the foraminiferans (Figure 2c). However, if the cumacean results from the Nordic Seas are included, then there is a much clearer poleward decline in species richness (not shown on this figure). This indicates that different taxonomic groups may, not surprisingly, exhibit different latitudinal trends, but studies have been few, and mechanisms to explain patterns remain to be tested.

A large-scale study undertaken in the UK Atlantic Margin highlights the difference in macrofaunal diversity between the Rockall Trough (Northeast Atlantic) and the Faroe-Shetland Channel (FSC), part of the Nordic Seas region [33], [112], [113]. Warm North Atlantic water is found in both areas; however the FSC also has colder waters (colder than 0°C) filling the deeper parts of the Channel. This significant thermal gradient along the western side of the FSC markedly influences both the diversity and distribution of the macrofauna [33], [112], [113]. The fauna in the warmer North Atlantic water is distinct from that in the colder Arctic waters. The Wyville-Thomson Ridge is a physical barrier that restricts the flow of colder water (but not completely, e.g., see [114]) into the Rockall Trough and Northeast Atlantic. Within the Channel there is a region at about 400 m water depth, potentially defined as an ecotone, of enhanced diversity where fauna from both the warm and cold waters can be found [33], [112], [113]. The HERMES program analyzed results from the European open slopes using the same sampling and identification protocols. Danovaro et al. [115] reported the highest biodiversity of nematodes was found at intermediate to high latitudes. However, these results need to be treated with caution because there is a lack of samples between 42° N and 70° N, and the analysis included results from the deep waters of both the Mediterranean and the Arctic, which may skew the results. Finally, in a study comparing point measurements of diversity at three deep-water locations along the European margin, Włodarska-Kowalczuk et al. [116] found little difference among infaunal diversity (Hurlbert's rarefaction) from the Greenland Sea (80° N), Rockall Trough (56° N), and Goban Spur (49° N) at depths below 1000 m, but two to four times lower diversity at the Arctic site between 1,000 and 3,000 m.

One of the biggest problems in trying to determine whether a latitudinal species-diversity gradient exists is that data are limited for the deep sea. Further, data are almost never collected for the specific use of investigating these gradients, and thus interpretation of results is somewhat difficult. There are also potential problems in making comparisons among the different studies, as there are often variations in the way that the fauna were collected, even within the same size groups. For example, the macrofauna may be collected with grabs, box corers, or megacorers as well as by using different diversity analyses [e.g., 103]. To make direct faunal comparisons among future studies, it is important that the sampling, level of identification, and analytical methods be standardized [117]. However, the many different habitats and environmental conditions of the deep sea make such standardization difficult. The different deep-water Census projects have tried to do this for their individual areas, but with limited success. The numerous different diversity analyses available also cause problems, as there are drawbacks to many of them and it is difficult to reach agreement on which to use.

#### Depth gradients

Water depth is a parameter that covaries with a variety of environmental characteristics, and not always in the same manner. This makes correlations of biodiversity (or any measurement) with depth open to considerable interpretation, because it is rarely depth per se (i.e., hydrostatic pressure) that is the cause of any relationship revealed. Despite this, depth is a useful, and easily measured, aggregate parameter that has been linked to spatial patterns in biodiversity. Over the range of ocean depths, data from a variety of invertebrate groups suggested a trend of increasing species richness to depths of about 2,500 m, and a subsequent decline at greater depths [reviewed in 52]. This has been corroborated in a study in the European Arctic Ocean [118], but the majority of recent empirical evidence from European waters points to either no change with depth [97], [119] or a monotonic (and sometimes exponential) decline with depth [91], [115], [119]–[123]. These studies have been performed in the Mediterranean, Norwegian, and Greenland Seas, and the Arctic Ocean, and faunal groups included foraminiferans, polychaetes, nematodes, crustaceans, and total macrofauna.

In many regional studies, latitude is a common covariate with depth. Depth gradually increases with latitude from about 20 m to 100 m for the area covered by the 1986 North Sea Benthos Survey (NSBS) [76], [77]. Earlier Glémarec [123] emphasized the importance of water temperature regime for species distributions in the North Sea. Due to stratification patterns in the North Sea, bottom temperature also often covaries with latitude and depth. The small annual temperature range (5–7°C) in the stratified central and northern North Sea, as compared to the southern North Sea (4–16°C), could explain why cold-water species are not found farther south than Dogger Bank (55°30′ N). By contrast, many southern “warm” species can survive the cold summer temperatures in the north. This mixture of cold- and warm-water species particularly in the deeper, northern parts of the North Sea could partly explain the latitudinal species richness trend, especially in the offshore areas.

**Figure 3:**
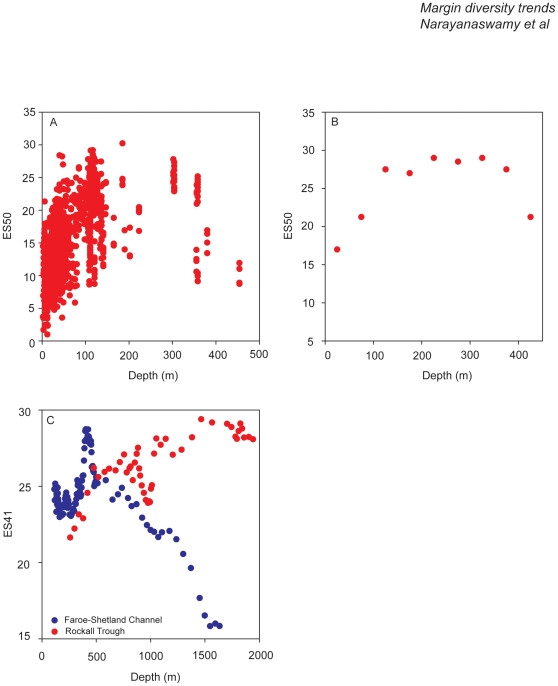
Variations in species diversity maxima with depth. (A) The ES50 for the North Sea soft-sediment benthos. The figure shown here excludes the results from the English Channel and therefore has been re-analysed and re-drawn to take this fact into account (see Rees et al. [84] for the full analysis). (B) The ES50 for the soft sediment European continental shelf macrofauna – mean values at 50 m depth bands (adapted from Renaud et al. [53]). (C) The ES41 for the deep Northeast Atlantic comparing macrofaunal results from North (Faroe-Shetland Channel) and South (Rockall Trough) of the Wyville-Thomson Ridge (data provided courtesy of BJ Bett [33]).

We have used the same North Sea dataset (as highlighted earlier) and have plotted the ES50 results against depth (Figure 3a) and find a strong positive relationship between depth and species diversity. Generally as depth increases there is an increase in diversity. Willems et al. [85] also found a significant relationship between average summer temperatures and ES50 in the full 2000 dataset. Reiss et al. [124] analyzed correlations between species richness and various environmental variables (e.g., depth, tidal and wave shear stress, salinity, mud content) and found that although many yielded significant correlation, the strongest (negative) correlation was with summer temperature. As highlighted above, determining which variable accounts for the trend in species richness is difficult, as most variables co-vary with one another. Jennings et al. [29], Zühlke et al. [93], and Callaway et al. [94] found that the epibenthic diversity was correlated with environmental variables (depth, sediment type, annual temperature range). Like Heip and Craeymeersch [77], Jennings et al. [29] point to temperature range as an important determinant for epibenthos species distributions in the North Sea, especially the contrast between stable conditions due to deep Atlantic inflow in the central and northern North Sea and variable inflow through the English Channel in the south. Zühlke et al. [93] found a positive correlation between diversity of free-living epifauna and depth, the latter being a proxy for temperature range and food availability in the North Sea. By contrast, diversity of sessile epifauna did not correlate well with depth, but in turn showed a strong (negative) correspondence with beam trawl effort. This could be interpreted as a causal relationship, that is, sessile species being particularly vulnerable to trawling. However, Callaway et al. [94] argue that distributions of commercial beam trawling and biotic variables, such as the presence or absence of sessile epibenthos, could result from similar environmental forcing (e.g., sediment type), without a direct causal relationship. A correlation analysis by Reiss et al. [124] with the 2000 data on epifauna diversity and a suite of environmental factors pointed to the importance of hydrographic factors (temperature range, salinity) and the insignificance of sediment type as a forcing factor.

At shelf depths, there is little theory to suggest how diversity should vary with depth, and again, multiple factors may covary with depth. These factors include light, disturbance, sediment grain size, current speed, oxygen concentration, salinity, and ecological interactions. In shallow waters (under 50 m), there is some evidence of depressed biodiversity among epifaunal and infaunal organisms in the shallowest areas, and increasing richness with depth thereafter (Figure 3b). Examples include epifauna in the Lena River delta [125], infauna in Norwegian fjords [126], and fouling organisms on stones in the Greenland Sea [127 (see other examples in regional sections below). It is highly likely that the proximal causes of the diversity patterns in these studies (e.g., sedimentation, scour) are different, however. In deeper waters of the continental shelf, several studies have shown an increase in richness to approximately 200 m depth followed by a decline to 300–500 m (Northern European polychaetes [119], Greenland shelf peracarid crustaceans [128], European-scale infauna [53]). It is likely that a different pattern exists for benthic macroalgae than for benthic invertebrates. A study by Middelboe et al. [129] noted the factors most responsible for predicting macroalgal diversity in Danish estuaries were salinity, water transparency, nutrient concentration, and availability of hard substrata. All of these may covary with water depth, but not always in predictable ways.

It is generally assumed that in the North Atlantic deep sea the relationship between macrofaunal diversity and depth fits a parabolic curve [59], [130]–[133], so that as depth increases so does diversity, reaching a peak at intermediate (bathyal) depths, before decreasing. Information for the Northeast Atlantic is still relatively sparse compared with that for the Northwest Atlantic (this is changing with the more recent programs of HERMES and COMARGE); however, it does still follow this general pattern with no further decline evident at abyssal depths [103], [134]. Along some areas of the European margin (55°–57° N), the parabolic relationship between diversity and depth is still evident, but with a maximum at a shallower depth of 1,400–1,800 m. Paterson and Lambshead [133] observed this trend for polychaetes on the Hebridean Slope, while Gage et al. [135] found that the macrofauna on the Scottish Continental Slope peaked in diversity at about 1,400 m water depth, and the lowest diversity was seen at 400 m. Using a large macrofaunal dataset (>300 stations) from the Faroe-Shetland Channel, a similar pattern is observed, however the peak in diversity occurs at a much shallower depth of about 450–550 m (Figure 3c) [33], [112], [113].

For the megafauna, peaks in diversity appear to vary depending on location and the taxonomic group being investigated. For example, Sibuet [136] found that asteroid diversity in the Bay of Biscay peaked at a depth of 2,200 m. More recent studies in the Porcupine Seabight and Porcupine Abyssal Plain have also found diversity in bivalves and asteroids to increase with increasing depth. However, the peaks in diversity were seen at 1,600 m and 1,800 m, respectively, followed by another peak in diversity at 4,100 m for bivalves [137] and 3,000–4,800 m for asteroids [138]. However, care must be taken when interpreting these results, as large numbers of single species were found at intermediate depths and will have skewed the picture toward describing bimodal peaks of diversity. Large numbers of the bivalve *Kelliella atlantica* (more than 90% of the individuals at 2,650 m) and of the asteroid *Hymenaster membranaceus,* also at a depth of about 2,600 m, lead to the noticeable decline in diversity at this depth [137], [138]. Olabarria [139] found that diversity of deep-sea prosobranch gastropods showed a unimodal distribution pattern with a decrease to about 1,600 m, followed by an increase to about 4,000 m. These results were opposite to those found by Howell et al. for seastars [138] and Olabarria for bivalves [137] where, as mentioned above, a bimodal distribution was seen. The potential problem with these results is that high abundance of one species, *Benthonella tenella,* in association with diversity indices that are size dependent, may have accentuated the decline in diversity between 1,400 and 1,600 m. However, the peak in diversity at about 4,000 m is similar to that found by Flach and de Bruin [134] for mollusks in the Porcupine Seabight and for seastars in the Porcupine Abyssal Plain [138].

Levin et al. [140] proposed a set of environmental factors that may be responsible for determining deep-sea diversity patterns, and these can in some cases be extended onto the shelf. Diversity is expected to increase with increasing food input, current speed, oxygen concentration of bottom waters, sediment heterogeneity, and disturbance. Then, at some point, diversity may continue to increase if heterogeneity increases, level off if oxygen concentration increases, or decline if the other three parameters continue to increase. These regional-scale factors may interact, and some may be more or less important at different sites. Indeed, productivity and food supply [63], [120], [121], [128], [141]–[143] and disturbance [127] have been cited as factors that may covary with depth and are more likely to be the proximal cause of observed diversity patterns. Despite this conceptual framework for understanding diversity patterns, uncertainty about how the factors interact at a given location limits its utility as a predictive tool for how diversity may be expected to vary with, for example, depth at a particular location, or over a continental or global scale. Instead, its value is in its consideration of valid ecological mechanisms on regional scales.

### Biodiversity in Arctic systems: Patterns in time and space

Whereas some of the discussion presented here has referenced studies performed in Arctic areas, the majority of Census activity related to the Arctic has been performed under ArcOD (Arctic Ocean Diversity: www.arcodiv.org), and will be reviewed elsewhere. Here we will, however, mention several general results especially pertinent to patterns of biodiversity in European Arctic waters.

It has long been held that Arctic diversity is low, and is considerably lower than Antarctic diversity [see e.g., [144]–[146]]. Although this is clearly true for some groups (e.g., brittle stars), recent studies suggest that this may not be a valid generalization (see discussion in [52]). In fact, early ideas may have been largely a consequence of low sampling effort. The last 10–15 years have seen increased research effort in both regions, a 20% increase in the number of benthic species cataloged in the Arctic [147], and the discovery that a large proportion of some elements of the Antarctic fauna are new to science (e.g., [148]). This, discovery of new species however, seems more likely to be the case for Antarctic rather than Arctic fauna, mainly because of the differences in geological history between the two regions. These results nevertheless underscore the continued need for description and recording of the biodiversity of these poorly studied regions.

Within the Arctic, there is a strong diversity gradient from east to west in the benthos of Arctic shelf regions. Whereas the Chukchi and East Siberian seas are home to about 950 species each, more than 1,080 species have been recorded from the Laptev Sea, 1,580 from the Kara Sea, and nearly 2,500 from the Barents Sea [149]. These findings are probably due to a variety of factors. Environmental conditions (depth, salinity, depth, sediment load, food input, hydrography) vary across the Eurasian Arctic and are probably responsible for part of this trend; but sampling effort also varies in a similar way. Additionally, and again related to the relatively short evolutionary history of the Arctic Ocean, the European Arctic fauna might be regarded as an impoverished subset of the Atlantic fauna. Hence, moving eastward into areas less influenced by Atlantic waters, species richness also declines. Two recent studies within the European Arctic have identified intriguing evidence of a difference in biodiversity between the eastern and western sides of the Greenland Sea. Kuklinski [127] reviewed the literature on benthic fauna inhabiting stones at depths of 0–50 m in the Greenland Sea and found a 30% higher species richness along the Greenland coast than along the Svalbard coast. In a small study of polychaete diversity, greater total species richness was found on the northeast Greenland shelf than in the central Barents Sea, and it took a sampling of more than 20 times the number of individuals in the Barents Sea to accumulate even this number of taxa [142]. These two areas differ in primary water masses, ice cover, and primary productivity, among other characteristics. More study is needed to determine the causes and general relevance of these results.

As in other world oceans, the North Atlantic and Arctic Ocean have seen pronounced variations in temperatures during the last 120,000 years [150]. However, as the North Atlantic and Arctic shelf sea are so much influenced by the GSS, variations in this current system (see e.g., [150], [151]) also seem to have a direct and detectable influence on the benthos in these areas. This may be exemplified through the past and current distribution of the blue mussel (*Mytilus edulis* L.) in the high Arctic (see [152] and references therein). Currently, the blue mussel has been recorded living on Svalbard, and its presence or absence on Svalbard during the last 11,000 years has been demonstrated to be linked directly to oscillations in ocean climate, as a result of changes in the volume transport of Atlantic water through the GSS [152]. Similar changes in both benthic and pelagic fauna have been detected and related not only to oscillations in the GSS at a longer time scale (e.g., [153]) but also to annual and decadal variations [154]. There is general agreement that the Arctic Ocean at present is in a transition toward a new, warmer state (e.g., [54]). The causes of such variations are not well understood, but variations in heat fluxes by means of the GSS are likely to have a massive influence on the benthos in the affected area.

### Changes in species richness along the Norwegian shelf

Norwegian naturalists and scientists have been sampling the marine inshore fauna along the Norwegian coast since the 1740s. The first publication containing information on marine species from the Norwegian coast is Bishop Erik Pontoppidan's work *Forsög paa Norges Naturlige Historie* published in Copenhagen in 1752–53. Some of the figures are detailed enough to be able to identify the species, for example, *Lophelia pertusa* (Linnaeus, 1758). The first Norwegian deep-sea sampling expedition, known as the Norwegian North Atlantic Expedition 1876-78, had sampling stations in some Norwegian fjords, the Norwegian shelf north of Bergen, the deep Norwegian Sea, the western Barents Sea and Svalbard ( =  Spitsbergen) up to 80° N. Since then, there have been no national benthic surveys conducted, and most of the data that have been collected have been associated with individual research projects and monitoring efforts around petroleum installations.

In 1995 a compilation of reliable information (museum collections, scientific literature and reports) on the presence of marine benthic macroorganisms was performed for the Norwegian coast [155]. Distributions of 3,950 species (algae 385 species, spermatophytes 12 species, invertebrates 3,409 species, and demersal fishes 144 species) were published in Brattegard and Holthe [156]. The original number of invertebrates has since been adjusted to 3,193 species because 118 species present in the Swedish Kosterfjord area (about 58°55′ N, 11°05′ E), close to the Norwegian border, had not actually been recorded in Norwegian waters, and 98 species were regarded as synonyms, following the most recent information from the World Register of Marine Species [157].

**Figure 4:**
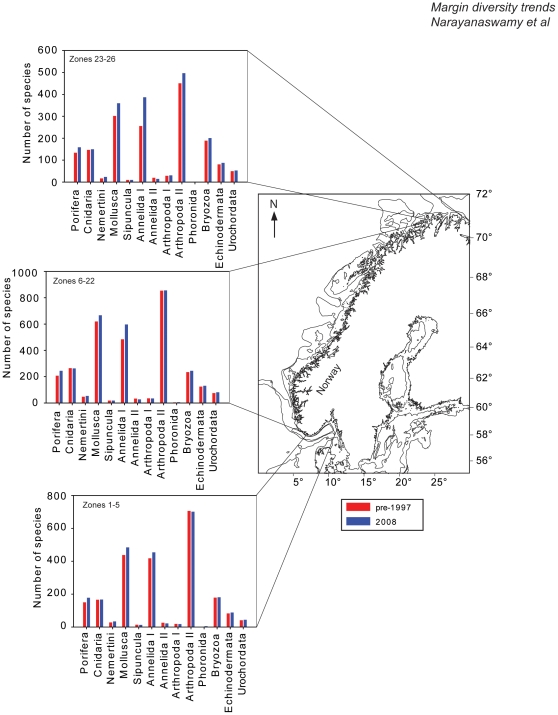
Changes in species richness along the Norwegian coast between 1997 and 2008. The Norwegian coast has been sub-divided into three regions [156]; zones 1–5  =  Skagerrak, zones 6–22  =  west Norway, zones 23–26  =  Finmark. Only the phyla which had changes in the number of species have been highlighted here. Annelida I  =  polychaetes; Annelida II  =  oligochaetes; Arthropoda I  =  pycnogonids; Arthropoda II  =  all other crustaceans.

Brattegard has created an updated dataset on the distribution of benthic invertebrates and included shifts in distribution range for many of the species (summarized in Table 2 and Figure 4). With these new results we are now able to determine what, if any changes there have been in species richness and species ranges. Since the late 1990's, one hundred and seventy additional species have been recorded in Norwegian waters since 1997. Of these, about 73% have their most northerly limits on the Norwegian coast and about 13% have their most southerly border on the Norwegian coast. Since the 1990s approximately 200 species have extended their northerly limits and are now found in Norwegian waters. During an 11-year timespan (1997–2008), there has been an 11% increase in the number of benthic species being recorded in new locations. The Norwegian coastline can be sub-divided into 3 regions (Figure 4) whereby zones 1–5 is the Skagerrak, zones 6–22 is West Norway and zones 23–26 is Finmark [156]. In the Finmark region, which is the most northerly there was 17% increase in the number of species recorded. The Skagerrak saw a ∼5% increase and a ∼7% increase was seen for Western Norway. A similar increase of about 9% has been noted for Svalbard alone during the same time period. In both cases, part of the increase may be due to more intensive sampling effort, but results are consistent with predicted impacts of climate warming.

**Table 2:** Comparison of species richness from 1997 and 2008.

Phylum	Species in sectors 1–5		Species in sectors 6–22		Species in sectors 23–26	
	Pre-1997	2008	Pre-1997	2008	Pre-1997	2008
Annelida I	416	**452**	482	**594**	254	**385**
Annelida II	24	**20**	31	**25**	18	**13**
Arthropoda I	17	**16**	33	**32**	27	**29**
Arthropoda II	704	**699**	851	**853**	449	**495**
Brachipoda	5	5	8	8	6	6
Bryozoa	177	179	232	**242**	187	**199**
Cephalochordata	1	1	1	1	0	0
Chaetognatha	1	1	1	1	0	0
Cnidaria	164	**165**	262	**260**	145	**148**
Echinodermata	81	**86**	122	**128**	79	**86**
Echiura	3	3	4	4	2	2
Hemichordata	4	4	3	3	0	0
Kamptozoa	18	18	20	20	6	6
Molluska	436	**482**	617	**664**	300	**358**
Nemertini	26	**32**	45	**51**	15	**22**
Phoronida	1	**3**	2	**3**	0	0
Porifera	148	**176**	205	**242**	132	**157**
Priapulida	2	2	3	3	3	3
Sipuncula	12	**11**	16	16	8	8
Urochordata	39	**42**	73	**79**	48	**51**
Xenoturbellida	0	0	1	1	0	0

The coast of Norway has been sub-divided into three regions [156]; zones 1–5  =  Skagerrak, zones 6–22  =  west Norway, zones 23–26  =  Finmark. Numbers in bold indicate an increase in the number of species. Annelida I  =  polychaetes; Annelida II  =  oligochaetes; Arthropoda I  =  pycnogonids; Arthropoda II  =  all other crustaceans; Bryozoa  =  Ectoprocta; Kamptozoa  =  Entroprocta.

### Anthropogenic impacts on diversity

There are several anthropogenic impacts thought to influence diversity in the North Sea, Arctic, and Northeast Atlantic, including fishing [158], activities by the oil and gas industry [33], [159], [160], eutrophication [161] and changes in climate [162], [163]. Currently there is limited routine monitoring undertaken to assess the changes in diversity in the Arctic and Northeast Atlantic. However, the potential for intense and diverse human impacts on the North Sea ecosystem has been recognized. Since 1986 a number of countries bordering the North Sea have initiated regular large-scale monitoring of the benthic infauna in their respective exclusive economic zones in the North Sea [78], which has given rise to a long time series [82]. More recently, efforts in monitoring benthic communities in coastal zones and offshore areas around the North Sea have increased in response to European regulations related to NATURA 2000 and the EU Water Framework Directives [164], [165].

It is well established that bottom trawling has a negative effect on benthic diversity, production, and community structure (e.g., [166], [167]). Bottom trawling can have a direct impact, causing mortality among the organisms living in the trawl path, especially epibenthic and fragile species, and among those species discarded after being caught in the nets [168]; it can also have indirect impacts due to physical destruction and resulting changes in habitat structure (e.g., [169], [170]). In the southern North Sea, the annual discard produced by beam trawlers, the dominant type of fisheries in the region was estimated to be 150,000–190,000 t of dead fish (mainly dab) and up to 85,000 t of dead invertebrates [75]. Recent maps showing the distribution of fishing intensity over the period 2000–2005 indicate that large parts of the area are fished 5–10 times per year [91]. However, in the North Sea it is difficult to discriminate the effects of beam trawling on biodiversity because of (1) lack of long time-series benthic studies before beam trawling began, (2) absence of controlled non-fished areas, (3) previously unregistered distribution of fishing effort, (4) occurrence of “nuisance” effects in the existing time series, such as eutrophication and climate change, and (5) the fact that benthos and fish distributions, and thus fisheries, are governed by the same environmental gradients, making it hard to prove causal relationships between changes in benthos and fishing.

Photographic surveys taken along the continental margin off the Hebrides have found that trawling is affecting the soft sediment and the fauna down to depths of 1,000 m. Trawling leaves large furrow marks in the sediment and causes smoothing of biogenic relief [171], which in turn affects the smaller fauna. At bathyal depths in the Faroe-Shetland Channel, rotting sponge remains have been found deep in a box-core sample [172]. The large enigmatic cold-water coral reefs formed by *Lophelia pertusa*, found in the North Sea, Norwegian Sea, Arctic region, and Northeast Atlantic, are particularly susceptible to damage by trawling. Seamounts are home to reef-forming organisms and large fish aggregations, and trawling is known to occur on them, leading to a reduction in faunal diversity and abundance. Growing concern has led to the protection of some large reef areas, such as the Sula Ridge and Røst reef off Norway [158], [173]. Other areas more recently protected include the “Darwin Mounds” and parts of the Rockall-Hatton Banks area.

There is limited regular monitoring on the impact that activity by the oil and gas industry has on the habitats and fauna of these regions, although Norway has a regular monitoring programme where the coastline is divided into regions which are sampled every three years. Research undertaken around Schiehallion and Foinaven fields north of Scotland found that megafaunal diversity was lowest in the immediate vicinity of the drilling area, where there was a lack of rare species, and that diversity increased at intermediate distances [160]. However, oil and gas platforms in the northern North Sea between 60 and 130 m have been found to provide a hard substrate for large epifauna, such as cold-water corals in regions where there is typically soft sediment [174]. The reefs can provide habitat for a species-rich epibenthos and fish community, which in many cases is not specifically associated with the coral itself, but benefits from the available hard substrate for settlement and its complex three-dimensional structures (e.g., [175]–[177]).

### Regime shifts and influence of changes in climate

Ongoing surveys since 1948 with the Continuous Plankton Recorder across the North Sea carried out by the Sir Alister Hardy Foundation for Ocean Science (SAHFOS) revealed periods with dramatic changes in the amount and composition of phyto- and zooplankton. One such period was in the late 1980s when a peak in phytoplankton color and oceanic zooplankton (*Calanus helgolandicus*) parallelled a rise in temperature and Atlantic inflow. In concert with this so-called regime shift [178], other biological variables (zooplankton composition, macobenthos biomass, fish and bird species) and physicochemical variables (oxygen, organophosphate, nitrate) changed simultaneously, or with a time lag. The increased Atlantic inflow causing the 1988 regime shift appears to be linked to higher flows in the slope current to the west of the British Isles, forced by far wind fields [179]. The permanent low-pressure system over Iceland and high-pressure system over the Azores largely controls the direction and strength of westerly winds into Europe. Variations in the strength and position of these systems are known as the North Atlantic Oscillation (NAO), and the anomalies are captured by the NAO index [180]. A high NAO index increases the degree of westerly winds and consequently milder temperatures over northern Europe, while a low NAO index usually associates with weaker westerly winds, allowing colder northerly winds to dominate over northern Europe. Hence, trends in the NAO index largely explain the variation in regional temperatures, precipitation, and the speed and direction of wind over the North Atlantic. The NAO trends have been found to mirror many aspects of the North Sea benthic ecosystem. Examples are the compositions of coastal and deeper benthic communities in the North Sea and Skagerrak [181]–[185], species richness in the Western Baltic [186], demersal fish recruitment [187], and the planktonic stages of benthic organisms [188].

### Conclusions

In this review, we present biodiversity data from infauna, epifauna, fish, and zooplankton from both regional and continental spatial scales, and from coastal habitats, continental shelves, and the deep-sea. This unprecedented collection of biodiversity findings can now be viewed more holistically such that general patterns and knowledge gaps can be identified, and recommendations for future research provided.

Within the North Atlantic and Arctic Ocean, we can see that benthic biodiversity does not seem to be correlated with latitude at a European continental scale. Rather, depth seems to be more important, as species richness generally increases to a depth of 200 m and then decreases to 300–500 m along the western European shelf. It is likely that trends over short and intermediate scales, and including some of the better known results such as from the North Sea, are similar to trends attributed to variation with depth - they actually represent coincident environmental gradients. In fact, where any regional scale latitudinal pattern has been observed, ecological explanations and coexisting gradients can be identified. In addition to water depth (itself a proxy for multiple causative factors), these include sediment parameters, salinity, temperature, and perhaps trawling pressure. Whereas the details of how variations in identified factors can produce diversity patterns requires further study, care needs to be taken when placing regional results into a latitudinal context, as this may hinder exploration of actual causal factors.

Whereas this may seem to be an academic question, it is important when evaluating the robustness of theory and in suggesting mechanisms responsible for the diversity patterns we observe. Renaud et al. [53] and Escaravage et al. [63] analyzed some of the largest datasets used for this purpose and found no evidence of an LSDG through the European seas for soft-sediment benthic communities. These studies span multiple ecological and physical gradients, and it is unlikely that their interpretations are confounded by any overarching gradient, beyond those used to explain LSDGs (e.g., solar radiation, glaciation history). It is important, however, to recognize that European waters extend southward only as far as 35° N, and much of the decline in diversity with latitude reported in some studies takes place in the tropics and subtropics (e.g. [61]). The European studies are valuable in the study of LSDGs in their testing of proposed mechanisms that are applicable for this range of latitude. Any mechanism predicting a broad decline in diversity with latitude between 35° N and 81° N (e.g., radiation following glaciation events on temperate and high latitude shelves), therefore, is not supported for soft-sediment benthos of the European continental margin.

Biodiversity is known to play a critical role in ecosystem function and, perhaps, system resilience [10], [189], [190]. Identifying mechanisms responsible for establishment and maintenance of biodiversity patterns is one of the most important scientific challenges if we are to manage marine ecosystems and their resources. Furthermore, climate change is expected to have its strongest effect in Arctic areas [10], as it did during the hemispherical warming period in the 1920s and 1930s, which was most strongly felt above 60° N [191], [192]. During this period, distributions of benthic and pelagic species showed dramatic northward shifts, especially on the western coasts of Greenland and Svalbard, and in the Barents Sea [193]–[195] and reviewed by Drinkwater [191]. The 5–17% increase since 1997 in species with their northern boundaries within Norwegian waters is evidence suggesting that similar range extensions are a result of the recent climatic warming period in European waters. The impact of increasing biodiversity in Arctic regions as a result of, increasing temperature and a regime shift towards a more boreal European Arctic is most likely a decrease in marine mammals and birds [196]. The effects of climate change on geographic distributions and population abundance of rocky shore fauna in Northern Europe over a 60-year time period were examined by Hawkins et al. [163]. They found that as the climate changed, there was a shift in dominance of sessile barnacle species from densely populating *Semibalanus* to slower growing chthamalids and that there was an increase in diversity of grazers such as limpets in northern parts of Europe [163]. These changes also affected the fucoid cover along the rocky shores, and such changes will no doubt have an impact on the biodiversity of these ecosystems, especially considering the numbers of sessile and mobile species living there [197]. Such climate-induced changes in distributional patterns, and recent findings of richness and diversity correlations with oceanic temperatures (for Barents Sea bivalves [198] and for fjord hard-bottom communities [153]), indicate the importance of viewing regional biodiversity as dynamic. This demands both that time-series data supplement spatially based biodiversity monitoring programs, and that a European perspective form the basis for any biodiversity management plan.

Mathematical/statistical modelling has not been widely used in biodiversity research but the studies that have been cited indicate value for identifying and perhaps explaining biodiversity patterns, for mapping hotspots or other specific areas for management purposes, and for predicting how system change due to climate variability or human activities may alter marine biodiversity. This can also help biodiversity researchers construct testable hypotheses from theory and ecological understanding, and allow us better understand mechanisms responsible for biodiversity patterns on different scales of time and space.

#### Gaps in knowledge

Throughout this paper, we have compiled and analyzed trends and patterns in biodiversity that have been observed and reported in the scientific literature. However, a comparative way of synthesizing and expressing the current state of knowledge is to identify and highlight gaps in knowledge. In which areas do we still lack vital knowledge? Based on the existing literature, spatial trends have been relatively well studied, however, there are still obvious gaps in knowledge related to e.g. large scale patterns in the hyperbenthos or general knowledge across habitat boundaries. Temporal trends, however, are scarce. Pending co-ordinated multi-national and possibly multi-decadal efforts that provide long time-series data and baseline values on which spatial variability may be interpreted; our understanding of the marine environment will remain limited. Furthermore, the organisms for which there exist time series data are predominantly either economically important or ecologically dominant species [199]. Consequently, even within relatively well studied geographical areas such as the North Sea, our current status of knowledge is severely restricted on two out of three potential dimensions; time, space and taxa. Potentially important aspects like the value of rare species for community resilience and structure is poorly understood, with obvious implications for proper management of marine resources.

To reduce our gaps in knowledge there is a need for greater coordination between European research programs, particularly in standardization of sampling techniques and analysis of data, for large-scale spatial and temporal patterns to be fully understood. Programs such as the Census of Marine Life and the EU-funded programs such as MarBEF and HERMES have instigated greater collaboration among researchers, and this should continue if we are to fully understand the influence that latitude, depth, and other environmental variables have on species diversity. Only then can we appreciate and comprehend the extent to which anthropogenic impacts, such as climate change and trawling, affect biodiversity.

## Supporting Information

File S1 Translation of the abstract into French, German, Italian and Spanish.
(0.04 MB DOC)

## References

[1] Worm B, Barbier EB, Beaumont N, Duffy JE, Folke C (2006). Impacts of biodiversity loss on ocean ecosystem services.. Science.

[2] Naeem S, Li S (1997). Biodiversity enhances ecosystem reliability.. Nature.

[3] Naeem S (2002). Ecosystem consequences of biodiversity loss: The evolution of a paradigm.. Ecology.

[4] Solan M, Raffaelli DG, Paterson DM, White PCL, Pierce GJ (2006). Marine biodiversity and ecosystem function: Empirical approaches and future research needs. Introduction.. Mar Ecol Prog Ser.

[5] Raffaelli D, Emmerson M, Solan M, Biles C, Paterson D (2003). Biodiversity and ecosystem processes in shallow coastal waters: an experimental approach.. J Sea Res.

[6] Solan M, Batty P, Bulling MT, Godbold JA (2008). How biodiversity affects ecosystem processes: implications for ecological revolutions and benthic ecosystem function.. Aqu Biol.

[7] Biles CL, Paterson DM, Ford RB, Solan M, Raffaelli DG (2002). Bioturbation, ecosystem functioning and community structure.. Hydrol Earth Syst Sci.

[8] Ieno EN, Solan M, Batty P, Pierce GJ (2006). Marine biodiversity and ecosystem function: empirical approaches and future research needs.. Mar Ecol Prog Ser.

[9] Ruesink JL, Feist BE, Harvey CJ, Hong JS, Trimble AC (2006). Changes in productivity associated with four introduced species: Ecosystem transformation of a pristine estuary.. Mar Ecol Prog Ser.

[10] Duffy, JE (2009). Why biodiversity is important to the functioning of real-world ecosystems.. Front Ecol Environ.

[11] Danovaro R, Gambi C, Dell'Anno A, Corinaldesi C, Fraschetti S (2008). Exponential decline of deep-sea ecosystem functioning linked to benthic biodiversity loss.. Curr Biol.

[12] Roberts DG, Hunter PM, Laughton AS (1979). Bathymetry of the Northeast Atlantic: continental margin around the British Isles.. Deep-Sea Res.

[13] Hansen B, Østerhus S (2000). North Atlantic - Nordic Seas exchanges.. Prog Oceanogr.

[14] Michaelsen W (1897). Polychaeta. Wissenschaftlichen Meeresuntersuchungen der Commission zur Wissenschaftlichen Untersuchung der deutschen Meere in Kiel.. Neue Folge.

[15] Stein U, Hukriede W, Rumohr H (1990). Historische Benthosdaten aus Nord- und Ostsee in den Jahren 1902–1912.. Mitt Zool Mus Univ Kiel.

[16] Künitzer A, Basford D, Craeymeersch JA, Dewarumez J-M, Dörjes J (1992). The Benthic Infauna of the North-Sea - Species Distribution and Assemblages.. ICES J Mar Sci.

[17] Gage JD (2001). Deep-sea benthic community and environmental impact assessment at the Atlantic Frontier.. Cont Shelf Res.

[18] Thomson JW (1873). The Depths of the Sea..

[19] Tyler PA (1986). Studies of a benthic time series: reproductive biology of benthic invertebrates in the Rockall Trough.. Proc Roy Soc Edinb Sect B.

[20] Gage JD (1986). The benthic fauna of the Rockall Trough: a regional distribution and bathymetric zonation.. Proc R Soc Edinb Sect B.

[21] Vanden Berghe E, Claus S, Appeltans W, Faulwetter S, Arvanitidis C (2009). MacroBen integrated database on benthic invertebrates if European continental shelves: a tool for large-scale analysis across Europe.. Mar Ecol Prog Ser.

[22] Ojaveer H, Jaanus A, MacKenzie BR, Martin G, Olenin S (2010). Status of biodiversity in the Baltic Sea.. PLoS ONE.

[23] Coll M, Piroddi C, Steenbeek J, Kaschner K, Lasram FBR (2010). The biodiversity of the Mediterranean Sea: Estimates, patterns, and threats.. PLoS ONE.

[24] Danovaro R, Company JB, Corinaldesi  C, D'Onghia G, Galil B (2010). Deep- Sea biodiversity in the Mediterranean Sea: the known, the unknown and the unknowable.. PLoS ONE.

[25] Eleftheriou A, McIntyre AD (2005). Methods for the study of marine benthos..

[26] Smith KL, Howard JD (1972). Comparison of a grab sampler and large volume corer.. Limnol Oceanogr.

[27] Dyer M F, Fry WG, Fry PD, Cranmer GJ (1982). A Series of North Sea benthos surveys with trawl and headline camera.. J Mar Biol Ass UK.

[28] Creutzberg F, Duineveld GCA, Vannort GJ (1987). The effect of different number of tickler chains on beam-trawl catches.. J Cons.

[29] Jennings S, Lancaster J, Woolmer A, Cotter J (1999). Distribution, diversity and abundance of epibenthic fauna in the North Sea.. J Mar Biol Ass UK.

[30] Reiss H, Meybohm K, Kröncke I (2006). Cold winter effects on benthic macrofauna communities in near- and offshore regions of the North Sea.. Helgoland Mar Res.

[31] Hessler RR, Sanders HL (1967). Faunal diversity in the deep sea.. Deep-Sea Res.

[32] Barnett PRO, Watson J, Connelly D (1984). A multiple corer for taking virtually undisturbed samples from shelf, bathyal and abyssal sediments.. Oceanol Acta.

[33] Bett BJ (2001). UK Atlantic Margin Environmental Survey: Introduction and overview of bathyal benthic ecology.. Cont Shelf Res.

[34] Gauthier O, Sarrazin J, Desbruyeres D (2009). Measure and mis-measure of species diversity in deep-sea chemosynthetic communities.. Mar Ecol Prog Ser.

[35] Gage JD, Hughes DJ, Vecino JLG (2002). Sieve size influences in estimating biomass, abundance and diversity in samples of deep-sea macrobenthos.. Mar Ecol Prog Ser.

[36] Degraer S, Moulaert I, Van Hoey G, Vincx M (2007). Sieving alive of after fixation: effects of sieving procedure on macrobenthic diversity, density and community structure.. Helgoland Mar Res.

[37] Blake JA, Narayanaswamy BE (2004). Benthic infaunal communities across the Weddell Sea Basin and South Sandwich Slope, Antarctica.. Deep Sea Res II.

[38] Gray JS (2000). The measurement of marine species diversity, with an application to the benthic fauna of the Norwegian continental shelf.. J Exp Mar Biol Ecol.

[39] Whittaker RJ, Willis KJ, Field R (2001). Scale and species richness: towards a general, hierarchical theory of species diversity.. J Biogeog.

[40] Gering JC, Crist TO (2002). The alpha-beta-regional relationship: providing new insights into local-regional patterns of species richness and scale dependence of diversity components.. Ecology Lett.

[41] Magurran AE (2004). Measuring biological diversity..

[42] Warwick RM, Clarke KR (1995). New “biodiversity” measures reveal a decrease in taxonomic distinctness with increasing stress.. Mar Ecol Prog Ser.

[43] Warwick RM, Clarke KR (1998). Taxonomic distinctness and environmental assessment.. J Appl Ecol.

[44] Warwick RM, Clarke KR (2001). Practical measures of marine biodiversity based on relateness of species.. Oceanogr Mar Biol Ann Rev.

[45] Price ARG, Keeling MJ, O'Callaghan CJ (1999). Ocean scale patterns of “biodiversity” of Atlantic asteroids determined from taxonomic distinctness and other measures.. Biol J Linn Soc.

[46] Clarke KR, Warwick RM (1999). The taxonomic distinctness measure of biodiversity: weighing up step lengths between hierarchical levels.. Mar Ecol Prog Ser.

[47] Hurlbert SH (1971). The non-concept of species diversity: A critique and alternative parameters.. Ecology.

[48] MarBEF (2004). European node of the Ocean Biogeographic Information System.. http://www.marbef.org/data/eurobis.php.

[49] Herman Y (1989). Arctic Seas: Climatology, Oceanography, Geology and Biology..

[50] Grebmeier JM, Barry JP (1991). The influence of oceanographic processes on pelagic-benthic coupling in polar regions: A benthic perspective.. J Mar Syst.

[51] Dayton PK, Mordida BJ, Bacon F (1994). Polar marine communities.. Am Zool.

[52] Piepenburg D (2005). Recent research on Arctic benthos: Common notions need to be revised.. Polar Biol.

[53] Renaud PE, Webb T, Bjørgesæter A, Karakassis I, Kedra M (2009). Continental-scale patterns in benthic invertebrate diversity: insights from the MarBEF database.. Mar Ecol Prog Ser.

[54] Greene CH, Pershing AJ, Cronin TM, Cecil N (2008). Arctic Climate Change and its impacts on the ecology of the North Atlantic.. Ecology.

[55] Arvanitidis C, Somerfield PJ, Rumohr H, Faulwetter S, Valavanis V (2009). Biological geography of the European Seas: results from the macrofaunal inventory of the soft-substrate communities.. Mar Ecol Prog Ser.

[56] Longhurst A (1998). Ecological geography of the Sea..

[57] Willig MR, Kaufman DM, Stevens RD (2003). Latitudinal gradients of biodiversity: Patterns, process, scale, and synthesis.. Annu Rev Ecol Syst.

[58] Hillebrand H (2004). On the generality of the latitudinal diversity gradient.. Am Nat.

[59] Rex MA (1981). Community structure in the deep-sea benthos.. Annu Rev Ecol Syst.

[60] Levinton J (1995). Marine biology, function, biodiversity, ecology..

[61] Macpherson E (2002). Large-scale species-richness patterns in the Atlantic Ocean.. Proc Roy Soc Lond B.

[62] Hillebrand H (2004). Strength, slope and variability of marine latitudinal gradients.. Mar Ecol Prog Ser.

[63] Escaravage V, Herman PMJ, Merckx B, Włodarska-Kowalczuk M, Amouroux J-M (2009). Distribution patterns of macrofaunal species diversity in subtidal soft sediments: findings on the biodiversity-productivity relation from a pan European dataset.. Mar Ecol Prog Ser.

[64] Somerfield PJ, Arvanitidis C, Faulwetter S, Chatzigeorgiou G, Vasileiadou A (2009). Assessing evidence for random assembly of marine benthic communities from regional species pools.. Mar Ecol Prog Ser.

[65] Colwell RK, Rahbek C, Gotelli N (2004). The mid-domain effect and species richness: what have we learned so far.. Am Nat.

[66] Hernandez-Stefanoni JL (2006). The role of landscape patterns of habitat types on plant species diversity of a tropical forest in Mexico.. Biodiv Cons.

[67] Thrush SF, Gray JS, Hewitt JE, Ugland KI (2006). Predicting the effects of habitat homogenization on marine biodiversity.. Ecol App.

[68] Verfaillie E, Van Lancker V, Van Meirvenne M (2006). Multivariate geostatistics for the predictive modeling of the surficial sand distribution in shelf seas.. Cont Shelf Res.

[69] Degraer S, Verfaillie E, Willems W, Adriaens E, Vincx M, Van Lancker V (2008). Habitat suitability as a mapping tool for macrobenthic communities: an example from the Belgian part of the North Sea.. Cont Shelf Res.

[70] Bekkby T, Rinde E, Erikstad L, Bakkestuen V (2009). Spatial predictive distribution modelling of the kelp species Laminaria hyperborea.. ICES J Mar Sci.

[71] Leichter JL, Witman JD, Witman JD, Kaustav R (2009). Basin scale oceanographic influences on marine macroecological patterns.. Marine Macroecology.

[72] Brown JH (1985). Macroecology..

[73] Gaston KJ, Blackburn TM (2000). Pattern and process in macroecology..

[74] Webb TJ, Aleffi F, Amouroux J-M, Bachelet G, Degraer S (2009). Macroecology of the European soft sediment benthos: insights from the Macroben database.. Mar Ecol Prog Ser.

[75] Lindeboom HJ, de Groot SJ (1994). Effects of beam trawling on the benthic fauna were initiated (IMPACT-1)..

[76] Heip C, Basford D, Craeymeersch JA, Dorjes J, Dewilde P (1992). Trends in biomass, density and diversity of North Sea macrofauna.. ICES J Mar Sci.

[77] Heip C, Craeymeersch JA (1995). Benthic community structures in the North-Sea.. Helgol Meeresunters.

[78] Duineveld GCA, de Wilde PAWJ, Kok A (1990). A synopsis of the macrobenthic assemblages and benthic ETS activity in the Dutch sector of the North Sea.. Neth J Sea Res.

[79] Ellingsen KE, Gray JS (2002). Spatial patterns of benthic diversity: Is there a latitudinal gradient along the Norwegian continental shelf?. J Anim Ecol.

[80] Hewitt JE, Thrush SF, Halliday J, Duffy C (2005). The importance of small-scale habitat structure for maintaining beta diversity.. Ecology.

[81] Huston M (1979). General hypothesis of species diversity.. Am Nat.

[82] Daan R, Mulder M (2006). The macrobenthic fauna in the Dutch Sector of the North Sea in 2005 and a comparison with previous data.. NIOZ-Rapport.

[83] Kröncke I (1992). Macrofauna standing stock of the Dogger Bank - a comparison 1950–54 versus 1985–87 - a final summary.. Helgol Meeresunters.

[84] Rees HL, Eggleton JD, Rachor E, Vanden Berghe E (2007). Structure and dynamics of the North Sea benthos.. ICES Cooperative Research Report.

[85] Willems W, Rees HL, Vincx M, Goethals P, Degraer S (2007). Relations and interactions between environmental factors and biotic properties. In: Rees HL, Eggleton JD, Rachor E,Vanden Berghe E, editors. Structure and dynamics of the North Sea benthos.. ICES Cooperative Research report.

[86] Craeymeersch JA, Witbaard R, Dijkman E, Meesters HWG (2008). Ruimtelijke en temporele patronene in de diversiteit van de macrobenthische infauna op het Nederlandse Continentaal Plat.. IMARES Report.

[87] Gray JS (2002). Species richness of marine soft sediments.. Mar Ecol Prog Ser.

[88] Warwick RM, Ashman CM, Brown AR, Clarke KR, Dowell B, Hart B, Lewis RE, Shillabeer N, Somerfield PJ, Tapp JF (2002). Inter-annual changes in the biodiversity and community structure of the macrobenthos in Tees Bay and the Tees estuary, UK, associated with local and regional environmental events.. Mar Ecol Prog Ser.

[89] Widdicombe S, Austen MC, Kendall MA, Olsgard F, Schaaning MT, Dashfield SL, Needham HR (2004). Importance of bioturbators for biodiversity maintenance: indirect effects of fishing disturbance.. Mar Ecol Prog Ser.

[90] Schroeder A (2005). Community dynamics and development of soft bottom macrozoobenthos in the German Bight (North Sea) 1969 – 2000. Ber Polarforsch Meeresforsch.

[91] Lindeboom HJ, Dijkman EM, Bos OG, Meesters EH, Cremer JSM (2008). Ecologische atlas Noordzee.. IMARES, Wageningen.

[92] Frauenheim K, Neumann V, Thiel H, Türkay M (1989). The distribution of larger epifauna during summer and winter in the North Sea and its suitability for environmental monitoring.. Senckenb Marit.

[93] Zühlke R, Alvsvåg J, de Boois I, Cotter J, Ehrich S, Kröncke I, Türkay M, Sündermann J (2001). Epibenthic diversity in the North Sea.. Burning issues of North Sea ecology. Proceedings of the 14th International Senckenberg Conference North Sea 2000. Senckenb Marit.

[94] Callaway R, Alsvag J, De Boois I, Cotter J, Ford A (2002). Diversity and community structure of epibenthic invertebrates and fish in the North Sea.. ICES J Mar Sci.

[95] Deubel H (2000). Structures and nutritional requirements of macrozoobenthic communities in the area of the Lomonosov Ridge in the Arctic Ocean.. Ber Polarforsch.

[96] Renaud PE, Ambrose WG, Vanreusel A, Clough LM (2006). Nematode and macrofaunal diversity in central Arctic Ocean benthos.. J Exp Mar Biol Ecol,.

[97] Bonsdorff E (2006). Zoobenthic diversity-gradients in the Baltic Sea: Continuous post-glacial succession in a stressed ecosystem.. J Exp Mar Biol Ecol.

[98] Kottelat M, Freyhof J (2007). Handbook of European Freshwater Fishes..

[99] Vassilenko SV, Hermann Y (1989). Arctic Ocean Cumacea.. The Arctic Seas: Climatology, Oceanography, Geology and Biology.

[100] Anisimova NA, Hermann Y (1989). Distributional patterns of echinoderms in the Eurasion sector of the Arctic Ocean.. The Arctic Seas: Climatology, Oceanography, Geology and Biology.

[101] Golikov, AN, Hermann Y (1989). Arctic Ocean gastropod prosobranchs.. The Arctic Seas: Climatology, Oceanography, Geology and Biology.

[102] Rex MA, Stuart CT, Hessler RR, Allen JA, Sanders HL (1993). Global-scale latitudinal patterns of species diversity in the deep-sea benthos.. Nature,.

[103] Rex MA, Etter RJ, Stuart CT, Ormond RFG, Gage JD, Angel MV (1997). Large-scale patterns of species diversity in the deep-sea benthos.. Marine Biodiversity.

[104] Rex MA, Stuart CT, Coyne G (2000). Latitudinal gradients of species richness in the deep-sea benthos of the North Atlantic.. Proc Nat Acad Sci, USA.

[105] Gage JD, Lambshead PJD, Bishop JDD, Stuart CT, Jones NS (2004). Large-scale biodiversity pattern of Cumacea (Pericarida: Crustacea) in the deep Atlantic.. Mar Ecol Prog Ser.

[106] Lambshead PJD, Tietjen JH, Ferrero TJ, Jensen P (2000). Latitudinal diversity gradients in the deep sea with special reference to North Atlantic nematodes.. Mar Ecol Prog Ser.

[107] Lambshead PJD, Brown Cj, Ferrero TJ, Mitchell NJ, Smith CR (2002). Latitudinal diversity patterns of deep-sea marine nematodes and organic fluxes: a test from the central equatorial Pacific.. Mar Ecol Prog Ser.

[108] Mokievsky V, Azovsky A (2002). Re-evaluation of species diversity patterns of free-living marine nematodes.. Mar Ecol Prog Ser.

[109] Thomas E, Gooday AJ (1996). Cenozoic deep-sea benthic foraminifers: tracers for changes in oceanic productivity?. Geology.

[110] Culver SJ, Buzas MA (2000). Global latitudinal species diversity gradient in deep-sea benthic foraminfera.. Deep-Sea Res.

[111] Corliss BH, Brown CW, Sun X, Showers WJ (2009). Deep-sea benthic diversity linked to seasonality of pelagic productivity.. Deep-Sea Res.

[112] Narayanaswamy BE, Bett BJ, Gage JD (2005). Ecology of bathyal polychaete fauna at an Arctic Atlantic boundary (Faroe-Shetland Channel, North-east Atlantic).. Mar Biol Res.

[113] Narayanaswamy BE, Bett BJ, Hughes DJ (2010). Deep-water macrofaunal diversity in the Faroe-Shetland region (NE Atlantic): A margin subject to an unusual thermal regime.. Mar Ecol.

[114] Sherwin TJ, Griffiths CR, Inall ME, Turrell WR (2008). Quantifying the overflow across the Wyville Thomson Ridge into the Rockall Trough.. Deep-Sea Res.

[115] Danovaro R, Canals M, Gambi C, Heussner S, Lampadariou N (2009). Exploring benthic biodiversity patterns and hotspots on European margin slopes.. Ocean.

[116] Włodarska-Kowalczuk M, Kendall MA, Węsławski JM, Klages M, Soltwedel T (2004). Depth gradients of benthic standing stock and diversity on the continental margin at a high-latitude ice-free site (off Spitzbergen, 79°N).. Deep-Sea Res.

[117] Stuart CT, Rex MA, Etter RJ, Tyler PA (2003). Large-scale spatial and temporal patterns of deep-sea benthic species diversity..

[118] Kröncke I (1998). Macrofauna communities in the Amundsen Basin, at the Morris Jesup Rise and at the Yermak Plateau (Eurasian Arctic Ocean).. Polar Biol.

[119] Dauvin J-C, Kendall M, Paterson G, Gentil F, Jirkov I (1994). An initial assessment of polychaete diversity in the northeastern Atlantic Ocean.. Biodiv Lett.

[120] Wollenburg JE, Kuhnt W (2000). The response of benthic foraminifers to carbon flux and primary production in the Arctic Ocean.. Marine Micropal.

[121] Kröncke I, Turkay M, Fiege D (2003). Macrofaunal communities in the eastern Mediterranean deep sea.. PSZNI Mar Ecol.

[122] Węsławski JM, Włodarska-Kowalczuk M, Legeżyńska J (2003). Occurrence of soft bottom macrofauna along a depth gradient in high Arctic, 79 °N.. Polish Polar Res.

[123] Glémarec M (1973). The benthic communities of the European North Atlantic continental shelf.. Oceanogr Mar Biol.

[124] Reiss H, Degraer S, Duineveld GCA, Kröncke I, Aldridge J (2010). Spatial patterns of infauna, epifauna, and demersal fish communities in the North Sea.. ICES J Mar Sci.

[125] Steffens M, Piepenburg D, Schmid MK (2006). Distribution and structure of macrobenthic fauna in the eastern Laptev Sea in relation to environmental factors.. Polar Biol.

[126] Holte B, Oug E, Cochrane S (2004). Depth-related benthic macrofaunal biodiversity patterns in three undisturbed north Norwegian fjords.. Sarsia.

[127] Kuklinski P (2009). Ecology of stone-encrusting organisms in the Greenland Sea - A review.. Polar Res.

[128] Brandt A, Vassilenko S, Piepenburg D, Thurston M (1996). The species composition of the peracarid fauna (Crustacea, Malacostraca) of the Northeast Water Polynya (Greenland).. Medd Grøn.

[129] Middelboe AL, Sand-Jensen K, Krause-Jensen D (2002). Patterns of macroalgal species diversity in Danish estuaries.. J Phycol.

[130] Rex MA, Rowe GT (1983). Geographical patterns of species diversity in the deep-sea benthos.. The sea, Vol. 8.

[131] Maciolek N, Grassle JF, Hecker B, Brown B, Blake JA (1987b). Study of biological processes on the U.S. North Atlantic slope and rise..

[132] Svavarsson J, Brattegard T, Stromberg J-O (1990). Distribution and diversity patterns of asellote isopods (Crustacea) in the deep Norwegian and Greenland Sea.. Prog Oceanogr.

[133] Paterson GLJ, Lambshead PJD (1995). Bathymetric patterns of polychaete diversity in the Rockall Trough, Northeast Atlantic.. Deep-Sea Res.

[134] Flach E, de Bruin W (1999). Diversity patterns in macrobenthos across a continental slope in the NE Atlantic.. J Sea Res.

[135] Gage JD, Lamont PA, Kroeger K, Paterson GLJ, Gonzalez-Vecino JL (2000). Patterns in deep-sea macrobenthos at the continental margin: Standing crop, diversity and faunal change on the continental slope off Scotland.. Hydrobiologia.

[136] Sibuet, M (1977). Repartition et diversité des echinoderms en zone profonde dans le Golfe de Gascogne.. Deep-Sea Res.

[137] Olabarria C (2005). Patterns of bathymetric zonation of bivalves in the Porcupine Seabight and adjacent Abyssal Plain, NE Atlantic.. Deep-Sea Res.

[138] Howell KL, Billett DSM, Tyler PA (2002). Depth-related distribution and abundance of seastars (Echinodermata: Asteroidea) in the Porcupine Seabight and Porcupine Abyssal Plain, N.E. Atlantic.. Deep-Sea Res.

[139] Olabarria C (2006). Faunal change and bathymetric diversity gradient in deep-sea prosobranchs from northeastern Atlantic.. Biodiv Cons.

[140] Levin LA, Etter RJ, Rex MA, Gooday AJ, Smith CR (2001). Environmental influences of regional deep-sea species diversity.. Annu Rev of Ecol Syst.

[141] Kröncke I (1994). Macrobenthos composition, abundance and biomass in the Arctic Ocean along a transect between Svalbard and the Makarov Basin.. Polar Biol.

[142] Ambrose WG, Renaud PE, Cochrane S, Denisenko SG, Skarðhamar J (2009). Polychaete diversity patterns on two Arctic shelves: Impacts of ice and primary production?. Zoosymposia.

[143] Cochrane SKJ, Denisenko SG, Renaud PE, Emblow CS, Ambrose WG (2009). Benthic macrofauna and productivity regimes in the Barents Sea - Ecological implications in a changing Arctic.. J Sea.

[144] Knox GA, Lowry JK, Dunbar MJ (1977). A comparison between the benthos of the Southern Ocean and the North Polar Ocean with special reference to the Amphipoda and the Polychaeta.. Polar Oceans.

[145] Dayton PK, Smith WO (1990). Polar Benthos.Smith WO, editor. Polar Oceanography.. Part B: Chemistry, biology and geology.

[146] Dunton KH (1992). Arctic biogeography: the paradox of the marine benthic fauna and flora.. Trends Ecol Evol.

[147] Sirenko BI (2001). List of species of free-living invertebrates of Eurasian Arctic seas and adjacent deep waters.. Explorations of the Fauna of the Seas, St Petersburg.

[148] Brandt A, Gooday AJ, Brandao SN, Brix S, Brokeland W (2007). First insights into the biodiveristy and biogeography of the Southern Ocean deep sea.. Nature.

[149] Brude OW, Moe KA, Bakken V, Hansson R, Larsen LH (1998). Northern Sea Route Dynamic Environmental Atlas..

[150] Rahmstorf S (2002). Ocean circulation and climate during the past 120,000 years.. Nature.

[151] Sarnthein M, Van Kreveld S, Erlenkreuser H, Grootes PM, Kucera M (2003). Centennial-to-millenial-scale periodicities of Holocene climate and sediment injections off the western Barents shelf, 75° N.. Boreas.

[152] Berge J, Johnsen G, Nilsen F, Gulliksen B, Slagstad D (2005). Ocean temperature oscillations enable reappearance of blue mussels *Mytilus edulis* in Svalbard after a 1000 year absence.. Mar Ecol Prog Ser.

[153] Beuchel F, Gulliksen B, Carroll ML (2006). Long-term patterns of rocky bottom macrobenthic cimmunity structure in an Arctic fjord (Kongsfjorden, Svalbard) in relation to climate variability (1980–2003).. J Mar Sys.

[154] Polyakov IV, Beszczynska A, Carmack EC, Dmitrenko IA, Fahrbach E (2005). One more step toward a warmer Arctic.. Geophys Res Lett.

[155] Brattegard T, Holthe T (1995). Distribution of marine, benthic macro-organisms in Norway..

[156] Brattegard T, Holthe T (1997). Distribution of marine, benthic macro-organisms in Norway: a tabulated catalogue..

[157] World Register of Marine Species (2009). http://marinespecies.org.

[158] Hall-Spencer J, Allain V, Fossa JH (2002). Trawling damage to Northeast Atlantic ancient coral reefs.. Proc Roy Soc Lond B.

[159] Gage JD, Roberts JM, Hartley JR, Humphery JD (2005). Potential impacts of deep-sea trawling on the benthic ecosystem along the Northern European continental margin: A review. In: Barnes BW, Thomas JP, editors. Benthic Habitats and the Effects of Fishing.. American Fisheries Society Symposium.

[160] Jones DOB, Wigham BD, Hudson IR, Bett BJ (2007). Anthropogenic disturbance of deep-sea megabenthic assemblages: A study with remotely operated vehicles in the Faroe-Shetland Channel, NE Atlantic.. Mar Biol.

[161] Claussen U, Zevenboom W, Brockmann U, Topcu D, Bot P (2009). Assessment of the eutrophication status of transitional, coastal and marine waters within OSPAR. 2^nd^ International Symposium on Research and Management of Eutrophication in Coastal Ecosystems Nyborg, Denmark, June 20-23, 2006.. Hydrobiol.

[162] Guinotte JM, Orr J, Cairns S, Freiwald A, Morgan L, George R (2006). Will human-induced changes in seawater chemistry alter the distribution of deep-sea scleractinian corals?. Front Ecol Env.

[163] Hawkins SJ, Moore PJ, Burrows MT, Poloczanska E, Mieszkowska N (2008). Complex interactions in a rapidly changing world: responses of rocky shore communities to recent climate change.. Clim Res.

[164] Management of Natura 2000 sites: Guidance (2009). http://ec.europa.eu/environment/nature/natura2000/management/guidance_en.htm.

[165] The EU Water Framework Directive – integrated river basin management for Europe (2009). http://ec.europa.eu/environment/water/water-framework/index_en.html.

[166] Kaiser MJ, Ramsay K, Richardson CA, Spence FE, Brand AR (2000). Chronic fishing disturbance has changed shelf sea benthic community structure.. J Anim Ecol.

[167] Kaiser MJ, Clarke KR, Hinz H, Austen MCV, Somerfield PJ (2006). Global analysis of response and recovery of benthic biota to fishing.. Mar Ecol Prog Ser.

[168] Bergman MJN, van Santbrink JW (2000). Mortality in megafaunal benthic populations caused by trawl fisheries on the Dutch continental shelf in the North Sea in 1994.. ICES J Mar Sci.

[169] Roberts JM, Wheeler AJ, Freiwald A (2006). Reefs of the deep: The biology and geology of cold-water coral ecosystems.. Science.

[170] Davies A, Wisshak M, Orr JC, Roberts JM (2008). Predicting suitable habitat for the cold-water coral Lophelia pertusa (Scleractinia).. Deep-Sea Res.

[171] Roberts JM, Harvey SM, Lamont PA, Gage JD, Humphery JD (2000). Seabed photography, environmental assessment and evidence for deep-water trawling on the continental margin west of the Hebrides.. Hydrobiologia.

[172] Bett BJ (2000). Benthic ecology of the Faeroe-Shetland Channel..

[173] Fosså J H, Mortensen PB, Furevik DM (2002). The deep-water coral *Lophelia pertusa* in Norwegian waters: distribution and fishery impacts.. Hydrobiologia.

[174] Gass SE, Roberts JM (2006). The occurrence of the cold-water coral *Lophelia pertusa* (Scleractinia) on oil and gas platforms in the North Sea: Colony growth, recruitment and environmental controls on distribution.. Mar Poll Bull.

[175] Jensen A, Frederiksen R (1992). The fauna associated with the bank-forming deepwater coral Lophelia pertusa (Scleractinaria) on the Faroe shelf.. Sarsia.

[176] Mortensen PB, Hovland M, Brattegard T, Farestveit R (1995). Deep water bioherms of the scleractinian coral *Lophelia pertusa* (L.) at 64° N on the Norwegian shelf: structure and associated megafauna.. Sarsia.

[177] Husebø Å, Nøttestad L, Fosså JH, Furevik DM, Jørgensen SB (2002). Distribution and abundance of fish in deep-sea coral habitats.. Hydrobiologia.

[178] Reid PC, Edwards M (2001). Long-term changes in the pelagos, benthos and fisheries of the North Sea.. Senckenb Marit.

[179] Reid PC, Edwards M, Beaugrand G, Skogen M, Stevens D (2003). Periodic changes in the zooplankton of the North Sea during the twentieth century linked to oceanic inflow.. Fish Oceanogr.

[180] Turrell JW (1995). Decadal trends in the North Atlantic Oscillation: regional temperatures and precipitation.. Science.

[181] Kröncke I, Dippner JW, Heyen H, Zeiss B (1998). Long-term changes in macrofaunal communities off Norderney (East Frisia, Germany) in relation to climate variability.. Mar Ecol Prog Ser.

[182] Hagberg J, Tunberg BG (2000). Studies on the covariation between physical factors and the long-term variation of the marine soft bottom macrofauna in Western Sweden.. Estuar Coast Shelf Sci.

[183] Rees HL, Pendle MA, Limpenny DS, Mason CE, Boyd SE (2006). Benthic responses to organic enrichment and climatic events in the western North Sea.. J Mar Biol Ass UK.

[184] Neumann H, Ehrich S, Kroncke I (2008). Effects of cold winters and climate on the temporal variability of an epibenthic community in the German Bight.. Clim Res.

[185] Frid CLJ, Garwood PR, Robinson L (2009). The North Sea benthic system: a 36 year time-series.. J Mar Biol Ass UK.

[186] Gröger J, Rumohr H (2006). Modelling and forecasting long-term dynamics of western Baltic macrobenthic fauna in relation to climate signals and environmental change.. J Sea Res.

[187] Dippner, JW (1997). Recruitment success of different fish stocks in the North Sea in relation to climate variability.. Dt Hydrogr Z.

[188] Kirby RR, Beaugrand G, Lindley JA (2008). Climate-induced effects on the meroplankton and the benthic-pelagic ecology of the North Sea.. Limnol Oceanogr.

[189] Palumbi SR, Sandifer PA, Allan JD, Beck MW, Fautin DG (2009). Managing for ocean biodiversity to sustain marine ecosystem services.. Front Ecol Environ.

[190] Walker MK, Thompson RM (2010). Consequences of realistic patterns of biodiversity loss an experimental test from the intertidal zone.. Mar Fresh Res.

[191] Drinkwater KF (2006). The regime shift of the 1920s and 1930s in the North Atlantic.. Prog Oceanogr.

[192] Renaud PE, Carroll ML, Ambrose WG, Duarte CM, Agusti S (2007). Effects of global warming on Arctic sea-floor communities and its consequences for higher trophic levels.. Impacts of warming on polar ecosystems.

[193] Blacker RW (1957). Benthic animals as indicators of hydrographic conditions and climatic change in Svalbard waters.. Fish Invest Ser.

[194] Nesis KN, Marty JJ (1960). Changes in the Barents Sea bottom fauna under the influence of fluctuations in the hydrographical regime (along the section on the Kola meridian)..

[195] Cushing DH (1982). Climate and Fisheries..

[196] Węsławski JM, Kwaśniewski S, Stempniewicz L (2009). Warming in the Arctic may result in the negative effects of increased biodiversity.. Polarforschung.

[197] Thompson RC, Wilson BJ, Tobin ML, Hill AS, Hawkins SJ (1996). Biologically generated habitat provision and diversity of rocky shore organisms at a hierarchy of spatial scales.. J Exp Mar Biol Ecol.

[198] Galkin YI (1998). Long-term changes in the distribution of molluscs in the Barents Sea related to the climate.. Ber Polarforsch.

[199] ICES (2008). Report of the ICES Advisory Committee, 2008..

