# Supplementary material for: Biodiversity Trends along the Western European Margin
Source: PLoS One. 2010 Dec 13;5(12):e14295. doi: 10.1371/journal.pone.0014295 (PMC3001438; doi:10.1371/journal.pone.0014295)
Supplement: File S1 — Translation of the abstract into French, German, Italian and Spanish. (0.04 MB DOC) [file pone.0014295.s001.doc]

**Supporting information: abstract translations**

# Biodiversity Trends along the Western European Margin

## Authors

Bhavani E Narayanaswamy^1^, Paul E Renaud^2, 4^, Gerard CA Duineveld^3^, Jørgen Berge^4^, Marc SS Lavaleye^3^, Henning Reiss^5^, Torleiv Brattegard^6^

^1^Scottish Association for Marine Science, Scottish Marine Institute, Oban, Argyll, UK

^2^Institute Akvaplan-niva AS, Polar Environmental Centre, Tromsø, Norway

^3^Netherlands Organisation for Scientific Research, Texel, the Netherlands

^4^The University Centre in Svalbard, Longyearbyen, Norway

^5^Department for Marine Research, Senckenberg Institute, Wilhelmshaven, Germany

^6^ Department of Biology, University of Bergen, Bergen, Norway

**Keywords:** Biodiversity, Northeast Atlantic, Arctic, North Sea, depth, latitudinal species-diversity gradient

**Correspondence:** Bhavani E Narayanaswamy, Scottish Association for Marine Science, Scottish Marine Institute, Oban, Argyll, PA37 1QA, UK

**E-mail:** Bhavani.Narayanaswamy@sams.ac.uk

Abstract translated into French:

**Variations de la biodiversité au large de l’Europe de l’ouest**

**Abstrait**

Les mers d’Europe occidentale couvrent à la fois une grande zone géographique et une grande variété d’habitats, et abritent plus de 10000 espèces de Métazoaires. Bien que des recherches aient été entreprises dans cette région depuis le début du XIX^ème^ siècle, beaucoup de nouvelles espèces et d’aires de répartition sont continuellement identifiées. Des études récentes, basées sur les plus grand jeux de données jamais utilisés en Europe, n'ont pu mettre en évidence de gradients latitudinaux de biodiversité dans l'endofaune subtidale à l'échelle du continent Européen. Le long du plateau ouest-Européen, la richesse en espèces augmente généralement jusqu’à 200 m de profondeur, puis décline à partir de 300-500 m. Dans les profondeurs de l’océan Atlantique Nord-Est, la diversité de la macrofaune suit une courbe unimodale quand la profondeur augmente. La mégafaune quant à elle semble suivre une distribution bimodale en fonction de la profondeur. Les résultats d’études régionales sont équivoques ; certaines études ou certains taxons suggèrent une diversité d’espèces croissante vers le pôle, d’autres non. Dans la mer du Nord, sans doute le système le mieux étudié d’Europe, il semble y avoir un gradient clair de biodiversité croissant avec la latitude. Ce dernier étant néanmoins occulté par des variations concomitantes de la profondeur et de l’intensité de pêche, il n’existe pas d’explication claire à ce patron de biodiversité. Des modifications liées au climat.des patrons de diversité et des aires de répartition, ont été récemment observées ; nous présentons ici des données originales sur les variations de la richesse en espèces des côtes norvégiennes sur les vingt dernières années, avec la région la plus septentrionale ayant enregistré une augmentation de 15% du nombre d’espèces découvertes.

Cette revue synthétise des données originales et publiées à différentes échelles spatiales et temporelles, de la côte aux abysses océaniques, afin de fournir un panorama des connaissances disponibles au large de l’Europe occidentale. Nous soulignons également les menaces pesant sur la biodiversité dans cette région, ainsi que les points où nos connaissances sont incomplètes.

Abstract translated into German:

**Biodiversitätstrends entlang des westeuropäischen Kontinentalabhangs**

**Zusammenfassung**

Die Meere entlang des westeuropäischen Kontinentalabhangs umfassen ein weites geographisches Gebiet mit zahlreichen unterschiedlichen Habitaten und sind Lebensraum von mehr als 10.000 Arten von Metazoa. Obwohl dieses riesige Gebiet schon seit dem frühen neunzehnten Jahrhundert erforscht wird, werden noch immer viele neue Arten beschrieben und neue Verbreitungsmuster aufgezeigt. Jüngere Studien, die die umgfangreichsten Datensätze nutzen, die jemals in derartigen europäischen Studien zum Einsatz gekommen sind, konnten, bezogen auf den gesamten Kontinent, keine breitengradabhängigen Trends in der Biodiversität der Schelf-Infauna finden. Entlang des westeuropäischen Kontinentalabhangs nimmt die Artenvielfalt generell bis zu einer Wassertiefe von 200 m zu, gefolgt von einer Abnahme ab 300-500 m. In größeren Tiefen des Nordostatlantik wird die Abhängigkeit der Makrofaunendiversität von der Wassertiefe durch eine unimodale Kurve beschrieben, während die Megafauna einem bimodalen Verteilungsmuster zu folgen scheint. Regionale Studien sind nicht eindeutig – einige zeigen polwärts in verschiedenen Gruppen eine Zunahme der Artenvielfalt, andere dagegen nicht. In der Nordsee, dem wohl am besten studierten europäischen Gewässersystem, scheint die Diversität mit zunehmendem Breitengrad deutlich zuzunehmen. Da dieser Trend aber durch ähnlich breitengradabhängige Gradienten in Tiefenverteilung und Trawling-Intensität überlagert wird, gibt es keine eindeutige Erklärung für dieses Diversitätsmuster. In jüngerer Zeit sind klimabedingte Änderungen in Diversitätsmustern und Verbreitungsgebieten beobachtet worden. Wir präsentieren bisher unveröffentlichte Daten zu Veränderungen im Artenreichtum, die in den letzten zwei Jahrzehnten entlang der norwegischen Küste beobachtet wurden. In dieser Zeit nahm in der nördlichsten Region die Anzahl der gefundenen Arten um mehr als 15% zu.

Diese Rezension verbindet bereits publizierte und neue Biodiversitätsdaten über multiple räumliche und zeitliche Skalen, von der Küste bis zur Tiefsee, um einen Überblick über das zu geben, was entlang des westeuropäischen Kontinentalabhangs bekannt ist. Daneben heben wir Gefahren für die Biodiversität der Region hervor und identifizieren bestehende Wissenslücken.

Abstract translated into Italian:

# Trend di biodiversità lungo i margini europei occidentali

# Estratto

I mari, lungo il margine occidentale Europeo coprono una vasta area geografica che comprende numerosi habitat diversi ed ospita oltre 10.000 specie di metazoi. Sebbene le ricerche scientifiche in questa vasta regione siano state effettuate a partire dal 1800, molte nuove specie sono ancora in via di descrizione e sono stati individuati nuovi modelli di distribuzione. Analisi recenti che incorporano la più ampia serie di dati mai utilizzati in Europa non sono riusciti a identificare chiari trend latitudinali di biodiversità dell’infauna della piattaforma continentale. La ricchezza di specie generalmente aumenta fino ad una profondità di 200 m, e quindi diminuisce fino a 300-500 m. Nel nord-est Atlantico profondo, una curva parabolica illustra come la diversità della macrofauna cambi combi con la profondità, mentre la megafauna sembra avere una distribuzione bimodale. Alcuni studi regionali identificano un aumento della diversità verso i poli, ma solo per alcuni taxa. Nel Mare del Nord, probabilmente il miglior sistema studiato nel margine Atlantico europeo, sembra esserci una tendenza ad un trend latitudinale di diversità. Dal momento che questa tendenza si confonde, simile gradienti latitudinali in profondità e l'intensità pesca a strascico, non esiste ancora una spiegazione chiara per il modello spaziale di biodiversità. Recentemente sono stati osservati shift legati ai cambiamenti climatici in grado di influenzare i modelli di distribuzione della diversità. In questo studio si riportano nuovi dati relativi alla biodiversità delle specie lungo la costa norvegese nel corso degli ultimi due decenni, dove la regione più settentrionale vede un aumento superiore al 15% nel numero di specie scoperte.

Questo lavoro sintetizza le informazioni relative a dati esistenti e nuovi dati sulla biodiversità attraverso scale spaziali e temporali multiple, dagli ambienti costieri a quelli profondi, per fornire una panoramica di ciò che è conosciuto lungo il margine Europeo occidentale. Si evidenziano anche le minacce alla biodiversità marine di questa regione, nonché le lacune ancora presenti nella nostra conoscenza sulla biodiversità marina in quest’area.

Abstract translated into Spanish:

**Tendencias de la biodiversidad a lo largo del margen de Europa occidental**

**Resumen**

Los mares europeos abarcan una amplia zona geográfica, numerosos hábitats diferentes, y son el hogar de más de 10.000 especies de metazoos. Aunque investigación en esta extensiva región se ha llevado a cabo desde principios de los años 1800, muchas nuevas especies son descritas y patrones de distribución identificados. Estudios recientes que utilizan la más extensa serie de datos que ha sido utilizada en estudios europeos no logran encontrar a escala continental tendencias latitudinales en la diversidad biológica de infauna de plataforma. A lo largo de la plataforma de Europa occidental, la riqueza de especies aumenta generalmente a una profundidad de 200 m, seguido por una disminución de 300 a 500 m. En el fondo del Atlántico Nordeste, una curva unimodal muestra cómo las especies de macrofauna cambian con la profundidad mientras que la megafauna parece tener una distribución bimodal. Los estudios regionales son equívocos, con aumentos hacia los polos en la diversidad de especies observadas en algunos estudios o taxones, pero no en otros. En el Mar del Norte, el sistema probablemente mejor estudiado en aguas europeas, parece haber una distintiva tendencia latitudinal en la diversidad. Debido a que esta tendencia es confundida por gradientes similares en profundidad y la intensidad de la pesca de arrastre, no hay una clara explicación para el patrón de biodiversidad. Cambios climáticos en los patrones de diversidad y variedad de especies se han observado recientemente. Aquí presentamos datos inéditos sobre los cambios en la riqueza de especies que se han observado a lo largo de la costa noruega en las últimas dos décadas, con la región más al norte con más de un 15% de aumento en el número de especies descubiertas allí.

Esta revisión sintetiza datos publicados y nuevos datos de biodiversidad a través de múltiples escalas espaciales y temporales, y desde la costa a la alta mar, para proporcionar una visión general de lo que se conoce a lo largo del margen de Europa Occidental. También resaltar las amenazas a la biodiversidad de la región, así como identificar donde todavía hay lagunas en nuestro conocimiento.
